# IRF-8/miR-451a regulates M-MDSC differentiation via the AMPK/mTOR signal pathway during lupus development

**DOI:** 10.1038/s41420-021-00568-z

**Published:** 2021-07-16

**Authors:** Guoping Shi, Dan Li, Dongya Zhang, Yujun Xu, Yuchen Pan, Li Lu, Jingman Li, Xiaoyu Xia, Huan Dou, Yayi Hou

**Affiliations:** 1 The State Key Laboratory of Pharmaceutical Biotechnology, Division of Immunology, Medical School, Nanjing University, Nanjing, 210093 PR China; 2 Department of Rheumatology and Immunology, Nanjing Drum Tower Hospital, The Affiliated Hospital of Nanjing University Medical School, Nanjing, 210008 China; 3 Jiangsu Key Laboratory of Molecular Medicine, Nanjing, 210093 PR China

**Keywords:** Autoimmunity, Lupus nephritis

## Abstract

Systemic lupus erythematosus (SLE) is a chronic systemic autoimmune disease. Myeloid-derived suppressor cells (MDSCs) have been found to be involved in the regulation of SLE development. However, little is known about the association between MDSC subsets and the factors that draw MDSCs into abnormal expansion. This study found that the percentage of M-MDSCs increased in mice with pristane-induced lupus. Toll-like receptor (TLR)7 signal activation and high interferon-α (IFN-α) level promoted M-MDSC differentiation in vitro. Moreover, both AMP-activated protein kinase (AMPK) agonist metformin and two mammalian targets of rapamycin (mTOR) inhibitors (INK128 and rapamycin) inhibited the percentage of M-MDSCs in lupus mice as well as in the TLR7- and IFN-α-induced bone marrow (BM) differentiation into MDSCs in vitro. In terms of mechanism, whole-genome transcriptome profiling was performed by RNA sequencing, revealing that the expression of the transcription factor IRF-8 was higher in M-MDSCs isolated from pristane-induced lupus mice, compared with control mice. IRF-8 was identified to be crucial for TLR7- and IFN-α-induced BM differentiation into MDSCs in vitro. Furthermore, interferon (IFN) regulatory factor8 (IRF-8) was targeted by miR-451a in M-MDSC differentiation. Of note, metformin-modified M-MDSCs could relieve lupus symptoms in pristane-induced lupus mice. The findings revealed a novel mechanism linking IRF-8/miR-451a to M-MDSC differentiation via the AMPK/mTOR signal pathway during lupus development. This study might provide an important reference for SLE therapy by targeting M-MDSCs.

## Introduction

Systemic lupus erythematosus (SLE) is a chronic systemic autoimmune disease characterized by ubiquitous autoantibody production, immune complex deposition, multiple-organ dysfunction, and aberrant tissue inflammation [1]. Several environmental, hormonal, and genetic factors play important roles during the development of SLE [2, 3]. Pristane (tetramethylpentadecane)-induced lupus is a murine model of SLE, facilitating research into the role of environmental factors in autoimmunity [4]. This murine lupus model is quite suitable for examining the links between dysregulated IFN-I production and the pathogenesis of human SLE. An increasing body of evidence suggests that early innate immune cells are highly important in the development of immune-mediated inflammation in SLE [5, 6]. Lupus flares are associated with a relative increase in the frequency of macrophages [7]. The frequency of inflammatory monocytes is also elevated in patients with SLE [8]. The number of plasmacytoid DCs (pDCs) decreases in the blood and accumulates in lesional skin or kidneys [9, 10]. Till 2010, the abnormalities of myeloid-derived suppressor cells (MDSCs) were found to be involved in the regulation of the innate immune response in autoimmune disorders [11].

MDSCs are a heterogeneous population of myeloid lineage cells derived from immature myeloid progenitors, which expand during chronic and acute inflammatory conditions [12, 13]. Murine MDSCs are characterized as CD11b^+^Gr1^+^ cells and can be typically divided into two subpopulations: G-MDSCs (CD11b^+^Ly6C^low^Ly6G^+^) and M-MDSCs (CD11b^+^Ly6C^+^Ly6G^-^) [12, 13]. Some findings on MDSCs in SLE are controversial [14–17]. A previous study showed that the frequencies of MDSCs were remarkably elevated in peripheral blood samples from patients with SLE, MRL/lpr mice, IMQ-lupus-prone mice, and mice with pristane-induced lupus [18–20]. In MRL/lpr lupus-prone mice, the splenic G-MDSCs expanded and positively correlated with disease severity [20]. The adoptive transfer of lupus MDSCs led to severe proteinuria and autoimmunity, impairing regulatory T(Treg) cells differentiation and promoting Th17 cell polarization [20]. Moreover, myeloid-derived CD11b^+^Gr1^+^ cells were confirmed to increase prior to abnormal changes in T and B cells during pristane-induced lupus development, and the mTOR pathway was critical for MDSCs in lupus development [21]. In mice with Toll-like receptor-7 (TLR7) agonist imiquimod-induced lupus, a significant expansion of MDSCs induced podocyte injury through increasing ROS in lupus nephritis (LN) [22]. These observations suggested that total MDSCs and their subsets played crucial roles in SLE development. Indeed, with heterogeneity among MDSCs and differential effects among subpopulations receiving much attention, understanding the abnormal differentiation mechanism of MDSC subtypes is crucial to reveal the role of MDSCs in SLE development.

Under normal physiological conditions, MDSCs are differentiated in bone marrow (BM) from hematopoietic progenitor cells and rapidly develop into neutrophils, monocytes, dendritic cells, and mature macrophages. Conversely, under pathological conditions, immature myeloid cells are expanded and converted into immunosuppressive MDSCs [23]. This implies that the differentiation of MDSCs is a complex and gradual phenomenon governed by multiple factors. IRF-8, a myeloid lineage-specific transcription factor, drives the differentiation of hematopoietic stem cells into granulocytes and macrophages via a distinct program [24–26]. Studies have confirmed that IRF-8 can regulate the differentiation of MDSCs in a variety of diseases, including breast cancer, colitis-associated colon cancer, and intestinal nematode infection [27–29]. In the pathogenesis of SLE, clinical large-scale data analysis showed that IRF-8 was closely related to the increased risk of SLE [30–34]. However, whether IRF-8 concentrated on the differentiation of MDSC subtypes in SLE development needs to be clarified.

A previous study showed that TLR7/IFN-α-mTOR signaling was significantly activated in total MDSCs in mice with early-aged lupus, suggesting that the abnormal differentiation of MDSCs might provide an important insight into the early diagnosis and treatment of SLE [21]. Moreover, this study provided evidence that INK128, a second-generation mTOR inhibitor, attenuated SLE by regulating total MDSCs [21]. Recently, the agonist for AMPK (upstream molecule of mTOR signal), metformin, was reported to relieve lupus symptoms by regulating abnormal T and B cells [35, 36]. Thus, it was hypothesized that metformin might improve lupus symptoms by regulating the differentiation of MDSCs during SLE development.

For a better understanding of the role of MDSC subtypes in SLE, the pristane-induced lupus murine model was used in the present study, characterized by high serum IFN-α levels and dysregulation of cellular immunity [21, 37, 38]. The changes in MDSC subtypes in mice under pristane induction were exhaustively detected. In vitro and in vivo experimental analyses were applied to determine whether AMPK/mTOR signaling was involved in the TLR7/IFN-α-prompted differentiation of M-MDSCs. In terms of mechanism, whole-genome transcriptome profiling was performed by RNA sequencing, trying to compare the differences between MDSCs in the normal physiological state and in SLE pathogenesis, as well as in the factors regulating the differentiation of MDSC subtypes. In all, the percentage of M-MDSCs significantly increased in mice with pristane-induced lupus, and AMPK/mTOR signaling was included in the differentiation of M-MDSCs. IRF-8 is crucial for TLR7/IFN-α-induced BM cell differentiation into M-MDSCs in vitro, being targeted by miR-451a. These findings indicated that metformin could regulate the differentiation of M-MDSCs precisely via IRF-8/miR-451a and have a potential therapeutic effect on lupus development.

## Results

### Both TLR7 signaling activation and IFN-α promoted the differentiation of M-MDSCs

**Fig. 1:**
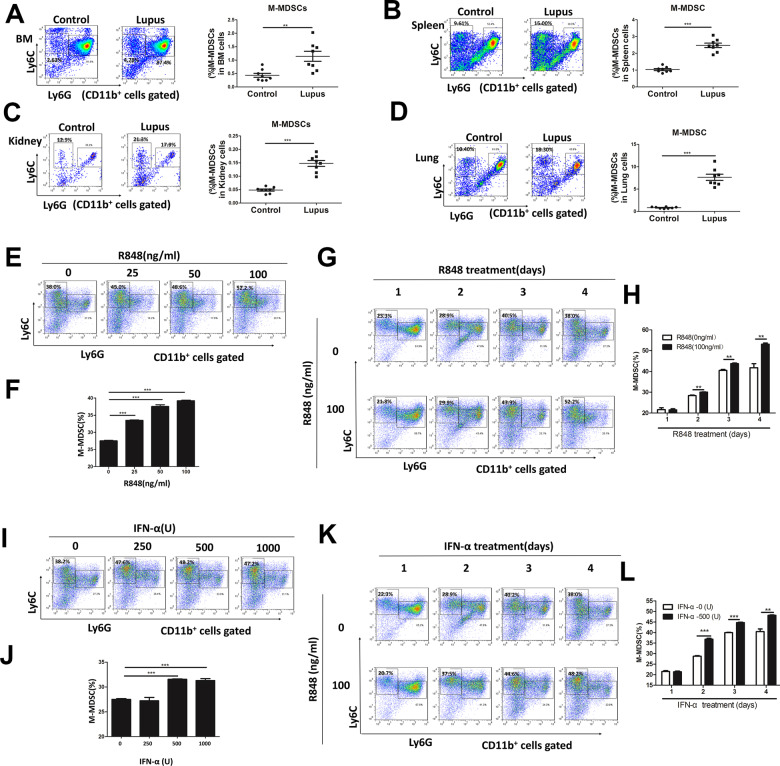
The percentage of M-MDSCs is increased in SLE development. BALB/c mice (10 weeks, *n* = 6–8/group) were given a single intraperitoneal injection of 0.5 ml pristane or PBS and monitored for 7 months. Representative flow cytometry analyses of M-MDSCs in bone marrow (BM) (**A**), spleen (**B**) kidney (**C**), and lung (**D**) in control mice and pristane-induced lupus mice. **E**, **F** Mouse BM cells were induced to MDSCs by adding GM-CSF (40 ng/ml) and IL-6 (40 ng/ml) and were treated with various concentrations of R848 (0–100 ng/ml). The percentage of M-MDSCs was determined by flow cytometry. **G**, **H** Mouse BM cells were treated with 100 ng/ml R848 on different days and were induced to MDSCs. The proportions of M-MDSCs analyzed by flow cytometry. **I**, **J** Mouse BM cells were induced to MDSCs by adding GM-CSF (40 ng/ml) and IL-6 (40 ng/ml) and were treated with various concentrations of IFN-α (0-1000 U). The percentage of M-MDSCs was determined by flow cytometry. **K**, **L** Mouse BM cells were treated with 500 U IFN-α on different days and were induced to MDSCs. The proportions of M-MDSCs were analyzed by flow cytometry. Data represent the mean scores ± SEM. All experiments were repeated three times. *P ≤ 0.05, **P ≤ 0.01, ***P ≤ 0.001.

The percentage of total MDSCs (CD11b^+^Gr1^+^ cells) was found to be elevated in the early stage of lupus progression [21]. To further study the role of MDSC subtypes (M-MDSCs and G-MDSCs), the changes in MDSC subtypes were detected in a pristane-induced lupus mouse model. As shown in Fig. 1, M-MDSCs increased in BM (Fig. 1A), spleen (Fig. 1B), kidney cells (Fig. 1C), and lung (Fig. 1D) in the lupus model mice compared with the control mice. On assaying the percentage of M-MDSCs in CD11b^+^ myeloid cells, we found that the percentage of M-MDSCs was elevated in BM (Fig. S1C), kidney cells (Fig. S1F), spleen (Fig. S1I), and lung (Fig. S1L). We also found that the percentage of G-MDSCs in total cells was elevated in BM (Fig. S1A), kidney cells (Fig. S1D), spleen (Fig. S1G), and lung (Fig. S1J), while the percentage of G-MDSCs reduced in CD11b^+^ myeloid cells in BM (Fig. S1B), kidney cells (Fig. S1E), spleen (Fig. S1H), and lung (Fig. S1K).

Next, the factors in the lupus microenvironment that could affect the differentiation of M-MDSCs were identified. Our previous study found that the activation of TLR7 signal and high IFN-α level was related to the differentiation of total CD11b^+^Gr1^+^ MDSCs [21]. However, the effect of TLR7 signal and IFN-α on M-MDSC is unclear [21]. To explore which MDSC subtypes were mainly affected in BM differentiation into MDSCs, BM cells were cultured in a medium supplemented with 40 ng/mL interleukin-6(IL-6) and 40 ng/mL granulocyte-macrophage colony-stimulating factor (GM-CSF) for 4 days. When stimulated with TLR7 agonist R848 in the culture, M-MDSCs increased (Fig. 1E–H) while G-MDSCs decreased (Fig. S1M, N) in a time- and dose-dependent manner. In addition, when stimulated with IFN-α, M-MDSCs (Fig. 1I–L) and G-MDSCs (Fig. S1O, P) also had similar effects. These results suggested that TLR7 and IFN-α in the lupus microenvironment might promote the differentiation of M-MDSCs in pristane-induced lupus mice.

Furthermore, to assay the variations in MDSC subtypes, M-MDSCs and G-MDSCs were respectively purified from spleens in mice with lupus and control mice using a MDSC isolation kit. Then, whole-genome transcriptome profiling was performed by RNA sequencing, and an EBseq algorithm was applied to filter the differentially expressed genes. First, differential mRNAs were found through mRNA-seq in M-MDSCs and G-MDSCs. Subsequently, the function of differential mRNAs was explored by pathway analysis, revealing that DNA replication pathway, Histidine metabolism pathway, Vitamin B6 metabolism pathway, Surfur relay system pathway, and the SLE signal pathway were significant differential pathways (TOP 25) in M-MDSCs (Fig. S2A, B). SLE signal pathway, Alcoholism pathway, Viral carcinogenesis pathway, Transcriptional misregulation in cancer pathway, and Cell cycle pathway were significant differential pathways (TOP 25) in G-MDSCs (Fig. S2A, B). We found that SLE signal pathway was a significantly different signaling pathway in both M-MDSC and G-MDSCs. Histone- and complement-related molecules in the SLE pathway changed significantly in M-MDSCs (Fig. S2C). Next, we analyzed the relationship between the most significant differential genes and TLR7 and IFN-α signaling pathways in M-MDSCs (Fig. S3A) and G-MDSCs (Fig. S3B). We found that TLR7/IFN-α signaling pathway plays a key role in regulating differential genes. This result suggested that the activation of TLR7/IFN-α signaling pathway in both M-MDSCs and G-MDSCs might play an important role in SLE development.

### AMPK regulated the differentiation of M-MDSCs in mice with pristane-induced lupus

A previous study found that mTOR inhibition could attenuate lupus symptoms by regulating the differentiation and functions of total CD11b^+^Gr1^+^ MDSCs. AMPK is an important upstream regulator of the mTOR signal. Metformin, as AMPK agonist, could inhibit mTOR by activating AMPK. Metformin relieved lupus symptoms by regulating abnormal T and B cells in a genetic background lupus mouse model [35, 36]. However, the therapeutic effect of metformin on environment factor pristane-induced SLE and the exact mechanism of action of metformin on the differentiation of M-MDSCs were unclear.

**Fig. 2:**
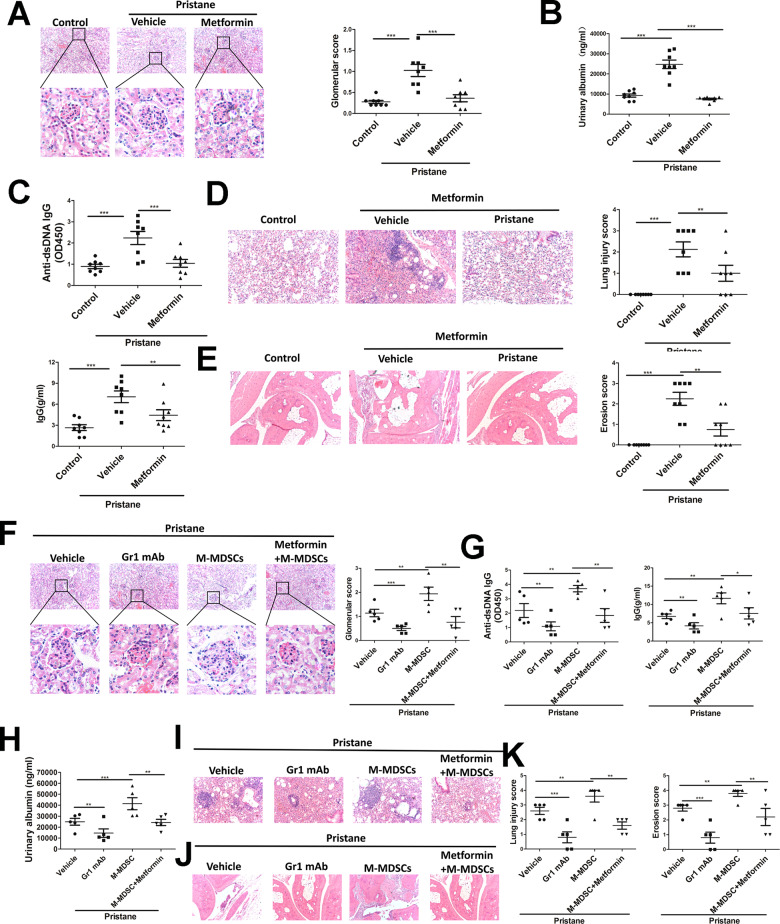
AMPK signal activation could attenuate lupus symptoms by regulating M-MDSCs differentiation in pristane-induced lupus mice. BALB/c mice (10 weeks, *n* = 6–8/group) were given a single intraperitoneal injection of 0.5 ml pristane or PBS and monitored for 7 months. Metformin-treated lupus mice for 2 months after 5 months pristane injection. **A** Kidney sections from each group showed histologic differences. **B** Proteinuria in each group was determined using Mouse Albumin ELISA Quantitation Set. **C** Serum levels of total IgG and IgG against dsDNA were determined by ELISA. **D** Lung sections from each group showed histologic differences. **E** Representative photographs of paws from each group. **F**–**K** Gr1mAb, M-MDSCs or Metformin-treated lupus M-MDSCs treated mice for 1 month. **F** Kidney sections from each group showed histologic differences. **G** Serum levels of total IgG and IgG against dsDNA were determined by ELISA Quantitation Set. **H** Proteinuria in each group was determined using mouse albumin. **I**, **K** Lung sections from each group showed histologic differences. **J**, **K** Representative histological sections of tarsal hind paw joints. Data represent the mean ± SEM. *P ≤ 0.05, **P ≤ 0.01, ***P ≤ 0.001.

First, to determine the effect of metformin on environment factor-induced SLE, pristane-induced lupus mice were treated with metformin for 2 months. As shown in Fig. 2A, kidneys from metformin-treated mice with lupus showed a better effect on glomerulonephritis and infiltration of lymphocytes compared with vehicle-treated mice with lupus. Metformin decreased proteinuria (Fig. 2B) and serum immunoglobin G (IgG) and anti-dsDNA IgG levels (Fig. 2C), which significantly increased in vehicle-treated mice with lupus. Metformin also gradually attenuated lung inflammation, which was severe in mice with pristane-induced lupus (Fig. 2D). In addition, metformin attenuated inflammatory infiltration and bone erosion in the tarsal joints of mice with lupus (Fig. 2E). These results together indicated that metformin had a therapeutic effect on environment factor pristane-induced SLE. The level of each MDSC subtype was quantified in the different tissues analyzed from metformin-treated SLE mice. We found that metformin significantly reduced the proportion of MDSCs subtypes, especially M-MDSCs (Fig. S4).

Next, to further explore whether the therapeutic effect of metformin on lupus was exerted by regulating M-MDSCs, four groups were set: control group, MDSC deletion group, lupus mice-derived M-MDSC adoptive transfer group, and metformin-treated M-MDSC adoptive transfer group. The effects of each group on lupus symptoms were tested 1 month later. The results showed that MDSC deletion could improve lupus symptoms, while the adoptive transfer of M-MDSCs derived from lupus mice could accelerate disease progression. Moreover, when metformin-treated M-MDSCs were infused into mice with lupus, lupus symptoms were in remission compared with the lupus mice-derived M-MDSC adoptive transfer group (Fig. 2F–K). Taken together, AMPK activation could attenuate lupus symptoms by regulating the differentiation of M-MDSCs in mice with pristane-induced lupus.

### M-MDSCs decreased by modulating the AMPK/mTOR signal pathway in vivo

**Fig. 3:**
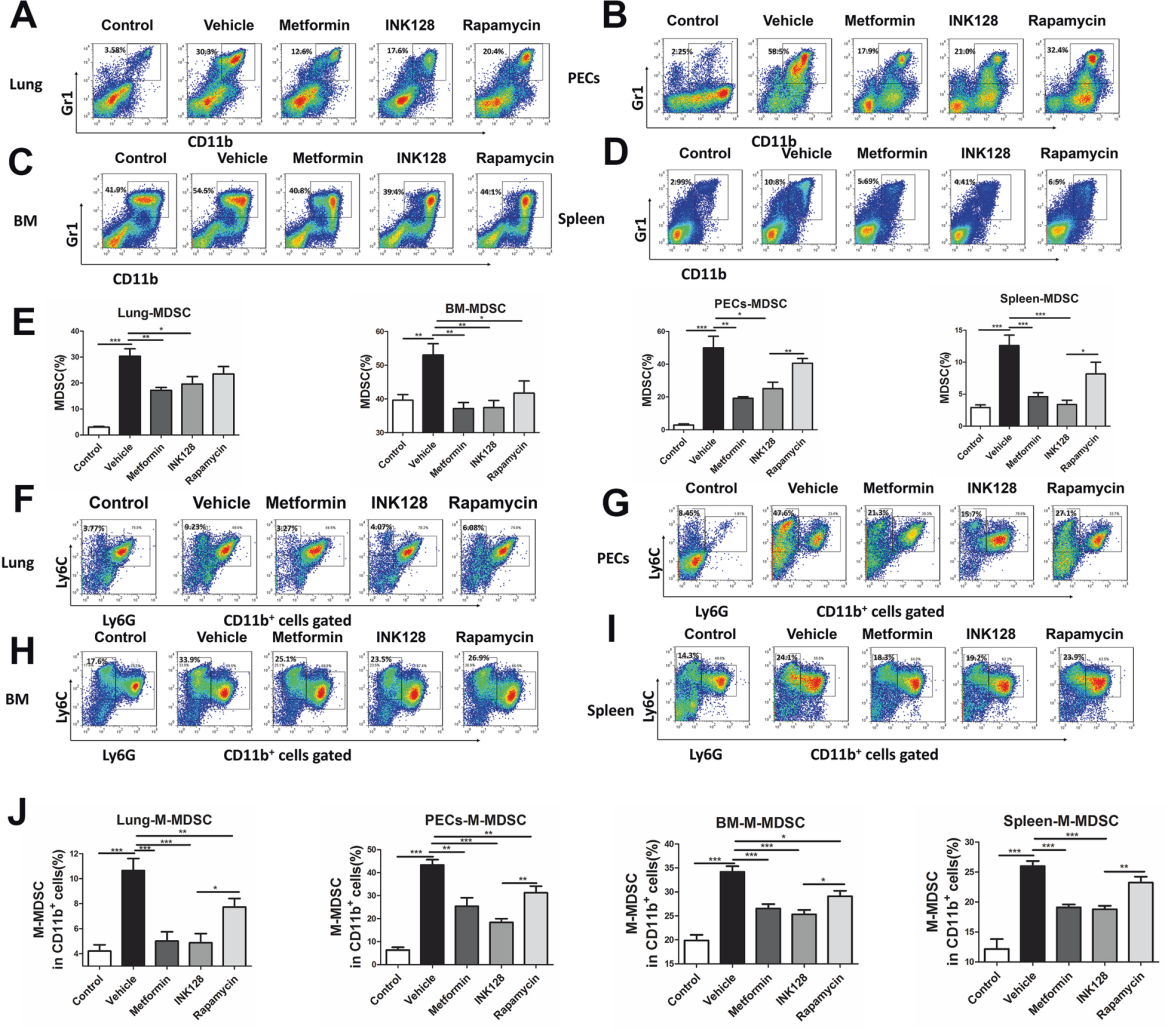
The percentage of M-MDSCs decreased by modulating AMPK/mTOR signal pathway in pristane-induced lupus mice. BALB/c mice (10 weeks, *n* = 6–8/group) were given a single intraperitoneal injection of 0.5 ml pristane or PBS, and the mice were treated with Metformin, INK128 or Rapamycin after 5 months pristane injection. After 2 months treatment, the proportions of total MDSCs and M-MDSCs were analyzed by flow cytometry. The percentage of CD11b^+^Gr1^+^ total MDSCs in lung (**A**), bone marrow (BM) (**B**), PECs (**C**), spleen (**D**). **E** The statistical graphs are shown. The percentage of M-MDSCs in lung (**E**), bone marrow (BM) (**F**), PECs (**G**), spleen (**H**). **I** The statistical graphs are shown. Data represent the mean ± SEM. *P ≤ 0.05, **P ≤ 0.01, ***P ≤ 0.001.

To confirm the role of the AMPK/mTOR pathway in M-MDSC expansion, BALB/c mice were injected with 0.5 mL of pristane. After 5 months, mice with lupus were treated with vehicle, AMPK agonist metformin, and mTOR inhibitors INK128 and rapamycin for another 2 months. As shown in Fig. 3A–E, mice with lupus treated with metformin, INK128, and rapamycin had a decreased percentage of total MDSCs in lungs (Fig. 3A), PECs (Fig. 3B), BM (Fig. 3C), and spleen (Fig. 3D) compared with vehicle-treated mice. The MDSC subtypes in mice with lupus treated with metformin, INK128, and rapamycin were further investigated. M-MDSCs in the lungs (Fig. 3F and J), peritoneal exudate cells (PECs) (Fig. 3G and J), BM (Fig. 3H and J), and spleen (Fig. 3I and J) of mice with lupus treated with metformin, INK128, and rapamycin compared with vehicle-treated mice. We found that metformin and mTOR inhibitors (INK128 and rapamycin) significantly inhibited the proportion of M-MDSCs in CD11b^+^ cells in lungs (Fig. 3F and J), peritoneal exudate cells (PECs) (Fig. 3G and J), BM (Fig. 3H and J), and spleen (Fig. 3I and J). In addition, both metformin and mTOR inhibitors can also increase the percentage of G-MDSCs in CD11b^+^ cells in lungs, peritoneal exudate cells (PECs), BM, and spleen (Fig. S1Q).

Furthermore, inhibiting the mTOR signal could reverse the abnormal expression of surface molecules (GO:001620) identified by Gene Onotology (GO) analysis among the differentially expressed genes and the SLE signal-related molecules (PATH ID:05322) identified by pathway analysis on M-MDSCs and G-MDSCs in mice with pristane-induced lupus (Fig. S5). Among the surface molecules, the expression of CD55, CD86, CD93, and CCR6 were decreased in G-MDSCs from pristane-induce lupus mice, and INK128 could increase the expression of these genes. In addition, we found that the expression of CD27, Anxa9, CCR9, and IL17Rβ were decreased in M-MDSCs from pristane-induce lupus mice, and INK128 could increase the expression of these genes. However, the expression of Anxa4 and TLR2 were increased in M-MDSCs, INK128 could inhibit Anxa4 and TLR2 expression in M-MDSCs in pristane-induced lupus mice. The differential expression of these surface molecules will help us to further differentiate more subtypes of MDSCs in SLE. And these results indicated that mTOR signal maybe a key pathway to regulate MDSCs in SLE development.

### AMPK/mTOR signal pathway regulated TLR7/IFN-α-induced M-MDSCs differentiation in vitro

**Fig. 4:**
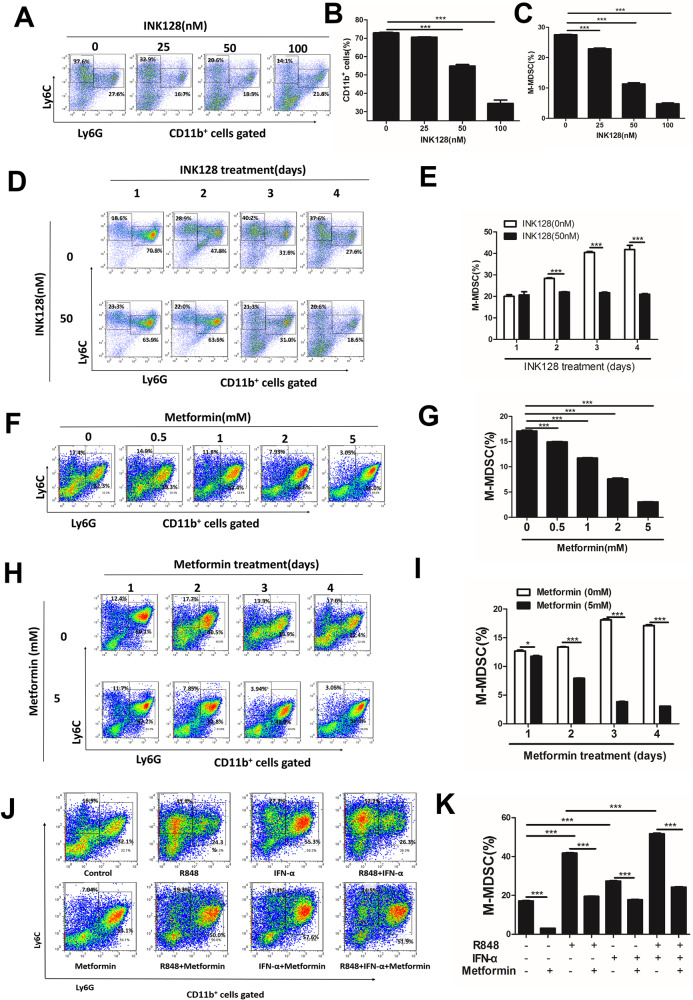
AMPK/mTOR signal pathway regulates TLR7/IFN-α-induced M-MDSCs differentiation in vitro. **A**–**C** Mouse BM cells were induced to MDSCs by adding GM-CSF (40 ng/ml) and IL-6 (40 ng/ml) and were treated with various concentrations of INK128 (0–100 nM). The percentage of M-MDSCs was determined by flow cytometry. **D**, **E** Mouse BM cells were treated with 50 nM INK128 on different days and were induced to MDSCs. The proportions of M-MDSCs and G-MDSCs were analyzed by flow cytometry. **F**, **G** Mouse BM cells were induced to MDSCs by adding GM-CSF (40 ng/ml) and IL-6 (40 ng/ml) and were treated with various concentrations of Metformin (0–5 mM). The percentage of M-MDSCs was determined by flow cytometry. **H**, **I** Mouse BM cells were treated with 2 mM INK128 with different days and were induced to MDSCs. The proportions of M-MDSCs were analyzed by flow cytometry. **J**, **K** Mouse BM cells were cultured for 4 days in GM-CSF and IL-6 with or without Metformin INK128, R848, IFN-α. Percentages of M-MDSCs were detected. Results were expressed as mean ± SD of three independent experiments. *P ≤ 0.05, **P ≤ 0.01, ***P ≤ 0.001.

To further explore the effect of the AMPK/mTOR signal on M-MDSC expansion, BM cells cultured in a medium supplemented with 40 ng/mL IL-6 and 40 ng/mL GM-CSF for 4 days. When the mTOR inhibitor INK128 was added to the culture, the percentage of M-MDSCs decreased (Fig. 4A–E) in a time-and dose-dependent manner. and the percentage of G-MDSCs also decreased (Fig. S6A, B). When the AMPK agonist metformin was added to the culture, M-MDSCs also decreased in a time- and dose-dependent manner (Fig. 4F–I). The changes of G-MDSCs affected by metformin are shown in (Fig. S6C–D). Our previous study found that the TLR7/IFN-α signal could activate mTOR in MDSCs. To investigate the role of the AMPK/mTOR signal pathway in the TLR7/IFN-α-induced differentiation of M-MDSCs, four groups were set: control group, R848 treatment group, R848, and Metformin treatment group, IFN-α treatment group, IFN-α, and metformin treatment group, R848 and IFN-α treatment group, and R848, IFN-α, and metformin treatment group. IFN-α/TLR7 signal promotes the differentiation of M-MDSCs in vitro, while metformin could inhibit these effects (Fig. 4J–K). The changes of G-MDSCs are shown in (Fig. S6E) and we found that IFN-α/TLR7 signal decreases the differentiation of G-MDSCs in vitro, while metformin could inhibit these effects. These results further indicated that the AMPK/mTOR signal pathway regulated the TLR7/IFN-α-induced differentiation of M-MDSCs in vitro.

### Transcription factor IRF-8 was crucial for the differentiation of M-MDSCs

**Fig. 5:**
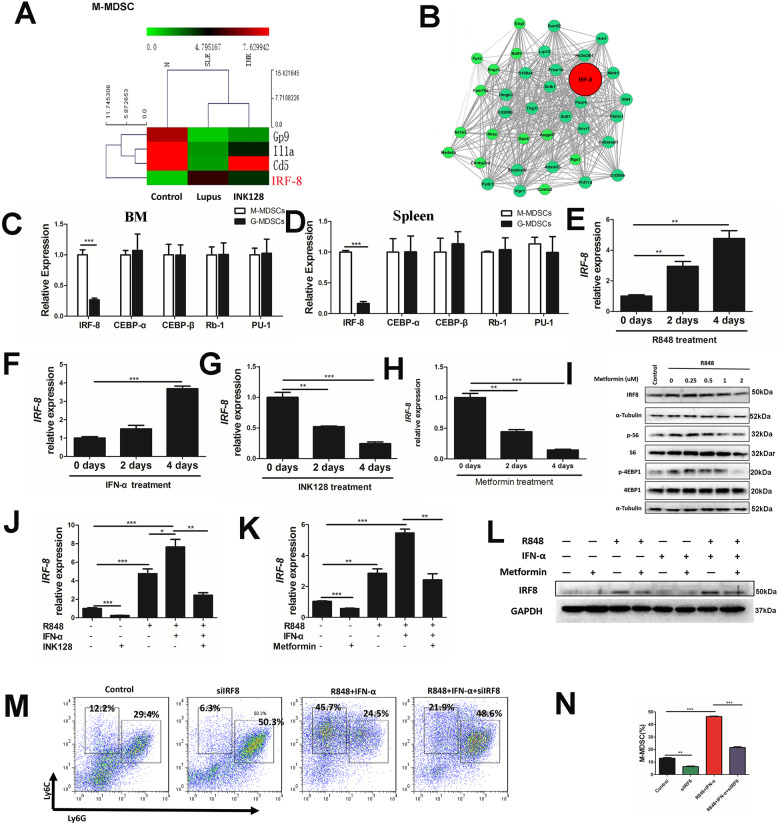
The transcription factor IRF-8 is crucial for M-MDSCs differentiation. After the Pristane-induced lupus mouse model was established, G-MDSCs and M-MDSCs were purified from Spleen-derived MDSCs mice using myeloid-derived suppressor cell isolation kit. Then, the whole-genome transcriptome profiling were performed by RNA sequencing. **A** The differential mRNA expression of hematopoietic cell lineage pathway in M-MDSCs between lupus mice and control mice are shown. **B** The relationship of IRF-8 with the differential mRNA expression in M-MDSCs are shown in Co-expression network relation map. Relative quantitation of *IRF-8, CEBP-β, CEBP-α, Rb1*, and *PU1* gene expressions by RT-PCR in between G-MDSCs and M-MDSCs in BM (**C**) and spleen (**D**) from lupus mice. Relative quantitation of *IRF-8* in BM cells treated with R848 (**E**), IFN-α (**F**), INK128 (**G**), and Metformin (**H**) in 0, 2, 4 d. **I** BM cells treated with 100 ng/ml R848 and different concentration of Metformin were at the same time. The expression of p-4EBP, 4EBP1, p-S6 and S6 was detected by western blot. **J** Relative quantitation of *IRF-8* in BM cells treated with R848, IFN-α or INK128 for 4 days. **K** Relative quantitation of *IRF-8* in BM cells treated with R848, IFN-α or Metformin for 4 days. **L** Mouse BM cells were cultured for 4 days in GM-CSF and IL-6 with or without Metformin, R848 and IFN-α. The expression of IRF-8 was detected by western blot. **M** Mouse BM cells were cultured for 4 days in GM-CSF and IL-6 with or without Metformin, R848 and IFN-α. The proportions of M-MDSCs were analyzed by flow cytometry. Results were expressed as mean ± SD of three independent experiments. *P ≤ 0.05, **P ≤ 0.01, ***P ≤ 0.001.

To explore the molecular mechanisms of the differentiation of M-MDSCs, the RNA sequencing results showed that the expression of IRF-8 in M-MDSCs from mice with lupus was higher than that in control mice (Fig. 5A). Meanwhile, the expression of IRF-8 in G-MDSCs from mice with lupus was lower than that in control mice (Fig. S7A, B). These results indicated that IRF-8 might play a crucial role in the differentiation of MDSC subtypes. To further explore the regulatory relationship between IRF-8 and AMPK/mTOR signal-related molecules, a co-expression analysis between IRF-8 and AMPK/mTOR signal-related molecules was conducted. IRF-8 was found to have a greater correlation with AMPK/mTOR signal-related molecules in M-MDSCs (Fig. 5B). The co-expression relationship of IRF-8 identified the significant roles in regulating MDSCs.

Next, to investigate whether IRF-8 was essential for the differentiation of MDSC subtypes, M-MDSCs and G-MDSCs were separately purified from spleens in mice with lupus and control mice using a MDSC isolation kit. Then, the expression of molecules related to myeloid cell differentiation in M-MDSCs and G-MDSCs in lupus development was compared. The expression of IRF-8 in M-MDSCs was found to be higher than that in G-MDSCs in both lupus BM-derived MDSCs (Fig. 5C) and spleen-derived MDSCs (Fig. 5D). Other myeloid cell differentiation molecules (CCAAT/enhancer-binding protein α (CEBP-α), CCAAT/enhancer-binding protein β (CEBP-β), Retino blastomal-1(Rb-1), and PU-1) were not changed. To identify the relationship between IRF-8 and AMPK/mTOR signal, BM cells were treated with R848, IFN-α, mTOR inhibitor INK128, and AMPK agonist metformin. R848 and IFN-α could elevate the expression of IRF-8 (Fig. 5E and F). However, the AMPK agonist metformin (Fig. 5H and I) and the mTOR inhibitor INK128 (Fig. 5G) could reduce the expression of IRF-8. We also detected the expression of p-AMPK (Fig. S8A, D), p-mTOR (Fig. S8B, D) and IRF-8 (Fig. S8C, D) in kidney in pristane-induced lupus mice and found that IRF-8 and mTOR were highly expressed in the kidney tissues of SLE mice, and INK128 and metformin could significantly inhibit the expression of IRF-8 and the phosphorylation of mTOR. In addition, p-AMPK was low expressed in kidney tissues of SLE mice, and INK128 and metformin significantly promoted the phosphorylation of AMPK (Fig. S8A–D). Moreover, IFN-α/TLR7 signal promotes the expression of IRF-8 in vitro, while the AMPK agonist metformin (Fig. 5K and L) and the mTOR inhibitor INK128 (Fig. 5J) could inhibit these effects. Furthermore, to identify whether IRF-8 was essential for the differentiation of M-MDSCs, BM cells were cultured in a medium supplemented with 40 ng/mL IL-6 and 40 ng/mL GM-CSF for 4 days. At the same time, R848, IFN-α, and siIRF-8 were added to the culture. IFN-α/TLR7 to promote the differentiation of M-MDSCs in vitro, while siIRF-8 could inhibit these effects (Fig. 5M, N). The effect of IRF8 on the differentiation of G-MDSCs are shown in (Fig. S7C). Taken together, these results indicated that the IRF-8 signal was crucial for TLR7/IFN-α-induced differentiation of M-MDSCs.

### miR-451a targeted IRF-8 in the differentiation of M-MDSCs

**Fig. 6:**
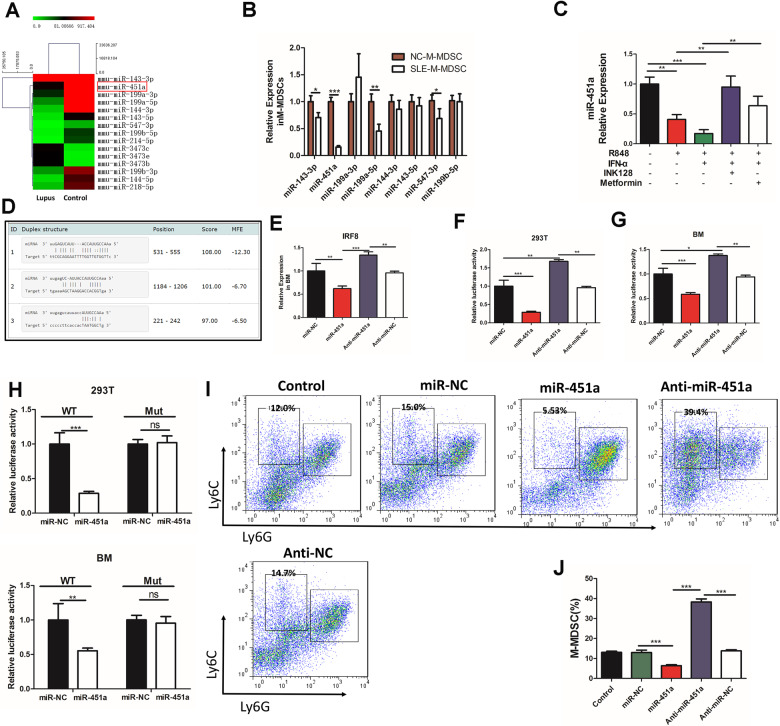
miR-451a targets the transcription factor IRF-8 in M-MDSCs differentiation. **A** The differential expression of microRNAs in M-MDSCs from Spleens of lupus mice and control mice is shown in the heat map. Relative quantitation of *miR-143-3p, miR-451a, miR-199a-3p, miR-199a-5p, miR-144-3p, miR-143-5p, miR-547-3p*, and *miR-199b-5p* expressions by RT-PCR in M-MDSCs. **B** from Spleens of lupus mice and control mice (*n* = 4). **C** Mouse BM cells were cultured for 4 days in GM-CSF and IL-6 with or without Metformin, R848 and IFN-α. The expression of *miR-451a* was detected by RT-PCR. **D** The target relationship between miR-451a and IRF-8 mRNA was predicted by miRTarBase website. **E** RT-PCR analysis of the effect of transfected miR-451a on IRF-8 expression compared with a negative control (miR-NC) in BM cells. **F** Luciferase reporter assays of the effect of miR-451a in 293T cells (**F**) and BM cells (**G**). Results are shown as mean ± SD from three independent experiments. *P < 0.05; **P < 0.01 compared with miR-NC control. **H** Luciferase reporter assay of the effect of miR-NC, miR-451a, anti-miR-451a, and anti-miR-NC in 293T cells and BM cells. Results are shown as mean ± SD of three independent experiments; *P < 0.05; **P < 0.01 compared with indicated control. **I** BM cells treated with miR-NC, miR-451a, anti-miR-451a, and anti-miR-NC. The proportions of M-MDSCs were analyzed by flow cytometry. Results were expressed as mean ± SD of three independent experiments. *P ≤ 0.05, **P ≤ 0.01, ***P ≤ 0.001.

miRNAs are small noncoding RNAs that regulate gene expression by targeting mRNAs in a sequence-specific manner by controlling degradation or inhibiting translation. The expression of specific miRNAs is involved in immune cells differentiation and function. In order to explore the role of mRNA in MDSCs (M-MDSCs and G-MDSCs) in SLE and the specific regulatory mechanism, we performed high throughput sequencing of miRNA. RNA-seq data on the differential expression of microRNAs in M-MDSCs from spleens of mice with lupus and control mice are shown in the heat map (Fig. 6A). We found that 12 microRNAs (miR-143-3p, miR-451a, miR-199a-3p, miR-199a-5p, miR-144-3p, miR-143-5p, miR-547-3p, miR-214-5p, miR-199b-5p, miR-199b-3p, miR-144b-5p, and miR-218-5p) are decreased and 3 microRNAs (miR-3473c, miR-3473e and miR-3473b) are increased. To further verify the accuracy of this result, the expression of microRNAs (miR-143-3p, miR-451a, miR-199a-3p, miR-199a-5p, miR-144-3p, miR-143-5p, miR-547-3p, and miR-199b-5p) on M-MDSCs by qPCR (Fig. 6B) and G-MDSCs (Fig. S9) from control mice and mice with lupus was detected. Further, IFN-α/TLR7 signal inhibits the expression of miR-451a. However, INK128 and metformin could inhibit these effects (Fig. 6C). Among the top 15 highly differentially expressed microRNAs, miR-451a was predicted to bind to IFR-8 mRNAs using the microRNA-targeted prediction software (miRTarBase website) (Fig. 6D). The reverse transcription-polymerase chain reaction (RT-PCR) analysis of BM cells showed that miR-451a could knock down the expression of IRF-8, efficiently (Fig. 6E). To determine whether miR-451a regulated the expression of IRF-8 through binding to the 3′-untranslated regions (3′-UTR) of IRF-8 mRNA, the entire 3′-UTR of IRF-8 mRNA containing the presumed miR-451a-binding sites was fused downstream of the firefly luciferase gene in a reporter plasmid. The resulting plasmid was transfected into 293T and BM cells along with miR-NC, miR-451a, anti-miR-451a, and anti-miR-NC RNA oligonucleotides. As expected, the luciferase reporter activity in cells transfected with miR-451a was reduced in 293T and BM cells compared with the cells transfected with the scrambled control (Fig. 6F, G). Then, a similar luciferase reporter assay was performed in both 293T and BM cells. The luciferase activity of the mutant reporter gene was not affected by the expression of miR-451a, whereas the activity of the wild-type reporter gene was markedly reduced (Fig. 6H).

To identify whether miR-451a was essential for the differentiation of M-MDSCs, BM cells were cultured in a medium supplemented with 40 ng/mL IL-6 and 40 ng/mL GM-CSF for 4 days. At the same time, miR-NC, miR-451a, anti-miR-451a, and anti-miR-NC were added to the culture in a single or mixed manner. Anti-miR-451a was found to promote the differentiation of M-MDSCs in vitro, while miR-451a could inhibit this differentiation (Fig. 6I, J). Taken together, these results indicated that the transcription factor IRF-8 was targeted by miR-451a in the differentiation of M-MDSCs.

## Discussion

The important roles of MDSCs have gained attention recently. However, different views exist on the role of MDSCs in SLE development [14–17]. The number of G-MDSCs elevated in male lupus-prone (NZB × NZW) F1 mice compared with age-matched female mice, directly suppressing B-cell activation and differentiation in vitro [14]. Laquinimod delayed LN manifestations by inducing the expansion of M-MDSCs and G-MDSCs in (NZB × NZW) F1 mice [15]. The infusion of MDSCs obtained from C57BL/6 mice resulted in an expansion of the regulatory B-cell population and an improvement in renal pathology in mice with roquin^san/san^ lupus [16].

In contrast, some studies explored the abnormal expansion of MDSCs in the pathogenesis of SLE. The number of M-MDSCs increased in the blood and skin samples of patients with cutaneous lupus compared with healthy controls, and the number of T cells reduced in a dose-dependent manner [17]. Some studies on the role of MDSCs in SLE development found that the percentages of MDSCs increased in patients with SLE, MRL/*lpr* mice, IMQ-lupus-prone mice, and mice with pristane-induced lupus [18, 20]. The percentage of MDSCs increased prior to the abnormal changes in Th17, Treg, T, and B cells during pristane-induced lupus development. TLR7/IFN-α-modified MDSCs promoted the imbalance of Th17/Tregs and were inclined to differentiate into macrophages via the mTOR pathway [21]. In the present study, G-MDSCs from diseased mice with lupus impaired Treg differentiation, and M-MDSCs promoted Th17-cell polarization [20]. Another study showed that MDSCs induced podocyte injury in LN via the ROS pathway [22]. The present study further investigated the role of MDSC subtypes in disease and found that the percentage of M-MDSCs increased in SLE development, and the activation of TLR7 signal and IFN-α in the lupus microenvironment jointly promoted the differentiation of M-MDSCs.

A previous study showed that TLR7/IFN-α-mTOR signaling was significantly activated in total MDSCs in mice with early-aged lupus, suggesting that the abnormal differentiation of MDSCs might provide an important insight into the early diagnosis and treatment of SLE [21]. Moreover, this study provided evidence that INK128, a second-generation mTOR inhibitor, attenuated SLE by regulating total MDSCs [21]. Recently, metformin, an AMPK agonist, was reported to relieve lupus symptoms by regulating abnormal T and B cells [35, 36]. The present study explored the treatment mechanism of metformin and found that metformin attenuated lupus symptoms by regulating the differentiation of M-MDSCs, and the percentage of M-MDSCs decreased by inhibiting the AMPK/mTOR signal pathway in vitro and in mice with pristane-induced lupus. Metformin could attenuate LN by reducing the glomerular injury in kidneys and reduce the inflammatory cell infiltration in joints and lungs by decreasing the differentiation of M-MDSCs. This might suggest that metformin could alleviate lupus symptoms by targeting and regulating the differentiation of M-MDSCs.

The RNA-seq results showed that the expression of surface molecules on M-MDSCs from lupus changed significantly compared with control mice. It indicated that the molecular phenotype of M-MDSCs changed during lupus development. This provided a reference for subsequent studies to find the molecular surface markers specific to MDSCs in lupus. When studying the signaling pathways of differentially expressed genes between M-MDSCs derived from mice with lupus and M-MDSCs derived from normal mice, the function of differentially expressed mRNAs was explored by pathway analysis, revealing that the SLE signal pathway (PATH ID:05322) was a significant pathway in both M-MDSCs and G-MDSCs. Among differentially expressed molecules, histone- and complement-related molecules in the SLE pathway changed significantly. This suggested that M-MDSCs present in lupus were different in phenotype and function under normal and pathological conditions. Future studies should explore the new functions of MDSCs during SLE development.

While further elucidating the molecular mechanisms involved in the differentiation of MDSCs in lupus, the expression of the transcription factor IRF-8 was found to be higher in M-MDSCs isolated from mice with pristane-induced lupus compared with control mice, with low expression in G-MDSCs. IRF-8, a myeloid lineage-specific transcription factor, drives the differentiation of hematopoietic stem cells into granulocytes and macrophages via a distinct program [24–26]. Studies confirmed that IRF-8 could regulate the differentiation of MDSCs in a variety of diseases [27–29]. The analysis of clinical large-scale data showed that IRF-8 was closely related to the increased risk of SLE, depleted the functional pDCs, and negatively regulated the expression of B cell-activating factor belonging to the TNF family (BAFF) in B cells in the pathogenesis of SLE [30–34]. The present study showed that the AMPK/mTOR signal affected the differentiation of M-MDSCs by regulating the expression of miR-451a/IRF-8.

Previous studies and the present study also found that the percentage of G-MDSCs was abnormal in mice with lupus. This suggested that G-MDSCs could play an important role in SLE development. Therefore, future studies should focus on finding effectors involved in promoting the abnormal differentiation and function of G-MDSCs. According to previous studies, AMPK-mTOR signaling pathway also plays a role in other immune cells during the pathogenesis of SLE, mainly in T cells. Our previous study found that abnormalities in the number and function of MDSCs occurred in the early stages of the disease, before the T cell abnormalities. Therefore, we believe that intervention targeting the abnormal number and function of MDSCs in the early stages of SLE may be a means of treatment for SLE. The role of MDSCs in the pathogenesis of SLE has been previously confirmed, so this study explored the relevant mechanism of AMPK-mTOR signaling pathway abnormality of MDSCs in SLE. Of course, MDSCs may not be the only target of Metformin and INK128 for the eventual mitigation of SLE, but, in the early stage of disease (before abnormalities occur in other cells), we believe that MDSCs may be important disease target cells for metformin and INK128. Through our previous study and the present study, it was found that TLR7/IFN-A mediated AMPK-mTOR signaling pathway has A more obvious regulatory effect on M-MDSCs than G-MDSCs in MDSCs subtypes. Therefore, in this study, the effect of this signaling pathway on M-MDSCs was discussed and studied. We believe that there may be other signaling pathways that play an important role in regulating the differentiation and function of G-MDSCs in SLE.

The present study found that the percentage of M-MDSCs increased in mice with pristane-induced lupus, and the TLR7 signal activation and the high IFN-α level could promote the differentiation of M-MDSCs in vitro. Next, the study showed that the AMPK agonist metformin and the mTOR inhibitors INK128 and rapamycin could reduce the percentage of M-MDSCs in mice with pristane-induced lupus and TLR7- and IFN-α-induced BM differentiation into MDSCs in vitro. To explore the mechanism, whole-genome transcriptome profiling was performed using RNA-seq, revealing that the expression of IRF-8 was higher in M-MDSCs in mice with pristane-induced lupus. This indicated that IRF-8 was crucial for TLR7- and IFN-α-induced BM differentiation into MDSCs in vitro. Also, IRF-8 was targeted by miR-451a in the differentiation of M-MDSCs. Furthermore, the study showed that metformin-modified M-MDSCs could relieve lupus symptoms in mice with pristane-induced lupus. This study helped understand the development of M-MDSCs and might provide an important reference for SLE therapy by targeting M-MDSCs.

## Materials and methods

### Antibodies and reagents

INK128, rapamycin, and metformin were purchased from Selleckchem Inc. Recombinant mouse IFN-α2, anti-CD11b-fluorescein isothiocyanate (FITC), anti-Gr1-allophycocyanin (APC), anti-Ly6G-phycoerythrin (PE), and anti-Ly6C-APC were purchased from Biolegend Inc. TRIzol reagent and SYBR green dye were purchased from Invitrogen Inc. Dulbecco’s modified Eagle’s medium (DMEM) and fetal bovine serum (FBS) were purchased from Gibco Inc. Collagenase type D and DNase I were purchased from Roche Inc. Pristane, *N*-methyl-2-pyrrolidone (NMP), and polyvinyl pyrrolidone (PVP) were purchased from Sigma Inc. Antibodies for α-tubulin (2144), p-S6 (4858S), S6 (2217S), p-4EBP-1 (2855S), 4EBP-1 (9644T), p-mTOR (5536T), p-AMPK (25375), and IRF-8 (83413T) were purchased from Signal Technology Inc. MDSC isolation kit, recombinant mouse IL-6, and GM-CSF were purchased from Miltenyi Biotec Inc. R848, CpG, poly I:C, and TNF-ɑ were purchased from Enzo Life Science Inc. Mouse albumin enzyme linked immunosorbent assay (ELISA) quantitation set, mouse anti-IgG, and anti-dsDNA IgG kit were purchased from Bethyl Laboratories Inc.

### Mice

Female BALB/c mice (6–8 weeks old) were obtained from Model Animal Research Center of Nanjing University (Nanjing, China) and housed in pathogen-free conditions in a 12-h light and dark cycle. All procedures involving mice were approved by the institutional license for animal care and use based on the Animal Care Committee at Nanjing University.

At 10 weeks of age, BALB/c mice (*n* = 6–8) received a single intraperitoneal (i.p.) injection of 0.5 mL of pristane or PBS. The mice received daily i.p. treatment with metformin, INK128, or rapamycin [1 mg/kg prepared in a 1-methyl-2-pyrrolidinone (NMP)/polyvinylpyrrolidone k30 (PVP) solution as described or vehicle (NMP/PVP solution) in the fifth month and administered to the mice 2 months later] [39]. Peritoneal cells, spleen, BM, kidney, and blood were harvested.

### MDSC depletion, isolation, and adoptive transfer experiment

At 10 weeks of age, BALB/c mice received a single intraperitoneal injection of 0.5 mL of pristane or PBS. After mice were treated with pristane for 5 months, 5-month-old BALB/c mice with pristane-induced lupus were injected i.p. with anti-Gr-1 antibodies (RB6-8C5, 200 μg; Biolegend, CA, USA) once every 4 days for another 2 months. For adoptive transfer, 2 × 10^5^ M-MDSCs were washed twice and resuspended in 200 μL of PBS and injected into mice via the tail vein weekly. To isolate MDSCs, tibias and femurs were removed from BALB/c mice, and BM cells were flushed. Then, BM cells were cultured in a medium supplemented with 40 ng/mL murine IL-6 and 40 ng/mL murine GM-CSF in the absence or presence of R848, IFN-ɑ, INK128, rapamycin, or metformin for 4 days. BM-derived MDSCs and spleen-derived MDSCs were purified from pristane-induced lupus mice using a MDSC isolation kit. G-MDSCs and M-MDSCs were purified from BM-derived MDSCs using the same kit.

### MDSC differentiation assay

BM cells were cultured in the presence of 40 ng/mL GM-CSF and IL-6 for 4 days. At the same time, R848 (0–100 ng/mL), IFN-α (0–1000 U), metformin (0–5 mM), INK128 (0–100 nM), and rapamycin (0–100 nM) were added to the culture medium. In some experiments, 100 ng/mL R848, 500 U IFN-ɑ, 2 mM metformin, 50 nM rapamycin, and 50 nM INK128 were incubated with 40 ng/mL GM-CSF and IL-6 for 4 days. After the different incubation periods, the cell phenotypes were determined by flow cytometry analysis.

### Cell culture, transfections, luciferase reporter assay, and siRNA interference assay

HEK293T cells were purchased from the Shanghai Institute of Cell Biology, Chinese Academy of Sciences (Shanghai, China). The cells were maintained at 37 °C in 5% CO_2_ in DMEM supplemented with 10% FBS (Invitrogen). Synthetic RNA, miR-451a mimic, miR-451a inhibitor (antisense-miR-451a), control mimics, and siRNA against IRF-8 were synthesized by GenePharma (Shanghai). The sequences used were as follows: miR-451a mimic, 5′-AAACCGUUACCAUUACUGAGUU-3′; miR-451a inhibitor, 5′-AACUCAGUAAUGGUAACGGUU-3′; IRF-8 siRNA, 5′-CCGGCAAGCAGGAUUACAATT-3′. HEK293 T cells and BM were transfected with oligonucleotides or indicated constructs using Lipofectamine 3000 (Invitrogen) according to the manufacturer’s protocols. To test the direct binding of miR-451a to the target gene *IRF-8*, a luciferase reporter assay (Promega) was performed. An shRNA sequence targeting IRF-8 cDNA was designed and synthesized by GenePharma.

### RNA sequencing

After the pristane-induced lupus mouse model was established, G-MDSCs and M-MDSCs were purified from spleen-derived MDSCs mice using the MDSC isolation kit. Then, whole-genome transcriptome profiling was performed by RNA sequencing (RNA-seq).

The RNA-seq was performed with the help of Novel Bioinformatics Co., Ltd (Shanghai, China). Total RNA was extracted using TRIzol reagent (Invitrogen), and the RNA quality was checked using Bioanalyzer 2200 (Aligent. The RNA was kept at –80 °C. The RNA with RNA integrity number (RIN) > 8.0 was right for cDNA library construction. Next, the cDNA libraries for single-end sequencing were prepared using an Ion Total RNA-Seq Kit v2.0 (Life Technologies). The cDNA libraries were then processed for the proton sequencing process according to the commercially available protocols, followed by the mapping of single-end reads. The clean reads were aligned to the mouse genome using the MapSplice program (v2.2.0). Moreover, the pathway analysis was performed, and the EBseq algorithm was applied to filter the differentially expressed genes. The significant analysis and FDR analysis were also performed. The RNA-seq data were analyzed using log2 fold change (log2FC > 1) and false discovery rate (FDR) (FDR > 0.05). Besides, gene co-expression networks were used to find the relationships among different mRNAs related to IRF-8.

### Flow cytometry analysis

The mice were sacrificed by cervical dislocation, the peritoneal cavity was lavaged with 3 mL of cold, sterile PBS, and the intraperitoneal fluid was harvested. The peritoneal cells were collected by centrifugation at 300 *g* for 10 min. BM cells were isolated as described previously by flushing femurs and tibiae. Single-cell suspensions of kidneys were prepared with collagenase type D (1 mg/mL) and DNase I (0.1 mg/mL) in Hank’s Balanced Salt Solution (HBSS) at 37 °C for 30 min. Then, the red cells from the kidneys were lysed. For cell surface marker staining, peritoneal cells, BM cells, splenocytes, Peripheral blood mononuclear cells (PBMCs), and lung and kidneys cells were prepared as single-cell suspensions. The cell suspensions were filtered through 70-µm cell strainers, and the lymphocytes were collected by centrifugation at 300 × *g* for 5 min at 4 °C. After washing, the cells were immediately prepared for flow cytometry. For the detection of mouse MDSC subsets, the cells were pre-incubated with FITC-conjugated anti-mouse CD11b mAb and APC-conjugated anti-mouse Gr1 mAb. For the detection of mouse G-MDSC and M-MDSC subsets, the cells were pre-incubated with FITC-conjugated anti-mouse CD11b mAb and PE-conjugated anti-mouse Ly6G mAb APC-conjugated anti-mouse Ly6C mAb. Then, the cells were stained for 30 min at 4 °C in the dark. After washing with the buffer, the cells were analyzed by flow cytometry. First, the CD11b^+^ cells were gated, and then, Ly6G and Ly6C markers were used to distinguish between G-MDSCs and M-MDSCs: G-MDSCs (CD11b^+^Ly6C^high^Ly6G^-^); M-MDSCs (CD11b^+^Ly6C^low^Ly6G^+^).

### Histological analyses

The sections were cut from paraffin-embedded tissues, fixed in formalin, and stained with hematoxylin and eosin.

### RNA extraction and quantitative real-time PCR

Total RNA was isolated using TRIzol reagent according to the manufacturer’s protocols. The real-time PCR assay was performed using SYBR green dye on the StepOne sequence detection system (Applied Biosystems, MA, USA). The relative abundance of genes was calculated using the 2^−ΔΔCT^ formula, and glyceraldehyde-3-phosphate dehydrogenase (GAPDH) was used as internal control. The primers can be found in supplementary material 2.

### Western blot analysis

The proteins were extracted using standard techniques [40]. The antibodies for IRF-8, p-S6, S6, p-4EBP-1, 4EBP-1, and horseradish peroxidase-conjugated anti-rabbit IgG for Western blot analysis were procured from Cell Signaling Technology (MA, USA). The protein bands were visualized using enhanced chemiluminescence (ECL) plus Western blotting detection reagents (Millipore, MA, USA). In the present study, α-tubulin was used as an internal control.

### Cytokine ELISA

Anti-IgG and anti-dsDNA IgG were analyzed using mouse anti-IgG and anti-dsDNA IgG Kit, and the sera were applied at dilutions of 1:100,000 and 1:300,000 according to the manufacturer’s protocols. Total urinary protein content was determined using a mouse albumin ELISA quantitation set (Bethyl Laboratories Inc.), and the urine was applied at dilutions of 1:100 according to the manufacturer’s protocols. The absorbance was determined using an ELx-800 Universal Microplate Reader (Bio-Tek, MA, USA).

### Statistical analysis

The results were expressed as mean ± standard error of mean (SEM) of three independent experiments, and each experiment included triplicate sets. The data were statistically evaluated using one-way ANOVA followed by Dunnett’s test between the control and multiple-dose groups. A *P* value <0.05 was indicated as a statistically significant difference.

## Supplementary information

Supplementary material 1

Marked up-Supplementary material 1

Supplementary material 2

FigureS1

FigureS2

FigureS3

FigureS4

FigureS5

FigureS6

FigureS7

FigureS8

FigureS9
