# Supplementary material for: IRF-8/miR-451a regulates M-MDSC differentiation via the AMPK/mTOR signal pathway during lupus development
Source: Cell Death Discov. 2021 Jul 16;7:179. doi: 10.1038/s41420-021-00568-z (PMC8289825; doi:10.1038/s41420-021-00568-z)
Supplement: Supplementary file 1 — Supplementary material 1 [file 41420_2021_568_MOESM1_ESM.docx]

**Figure S1.** **The percentage of MDSCs changed by modulating TLR7/IFN-α-AMPK/mTOR signal pathway in pristane-induced lupus mice.** BALB/c mice (10 weeks, n=6-8/group) were given a single intraperitoneal injection of 0.5 ml pristane or PBS and monitored for 7 months. Representative flow cytometry analyses of G-MDSCs in bone marrow (BM) **(A)**, Spleen **(D)** Kidney **(G)** and Lung **(J)** in control mice and pristane-induced lupus mice. Representative flow cytometry analyses of G-MDSCs and M-MDSCs in CD11b^+^cells in bone marrow (BM) **(B-C)**, Spleen **(E-F)** Kidney **(H-I)** and Lung **(K-L)** in control mice and pristane-induced lupus mice. **(M)** Mouse BM cells were induced to MDSCs by adding GM-CSF (40ng/ml) and IL-6 (40ng/ml) and were treated with various concentrations of R848 (0-100ng/ml). Percentage of G-MDSCs were determined by flow cytometry. **(N)** Mouse BM cells were treated with 100ng/ml R848 with different days and were induced to MDSCs. The proportions of G-MDSCs analyzed by flow cytometry. **(O)** Mouse BM cells were induced to MDSCs by adding GM-CSF (40ng/ml) and IL-6 (40ng/ml) and were treated with various concentrations of IFN-α (0-1000U). Percentage of M-MDSCs were determined by flow cytometry. **(P)** Mouse BM cells were treated with 500U IFN-α with different days and were induced to MDSCs. BALB/c mice (10 weeks, n=6-8/group) were given a single intraperitoneal injection of 0.5 ml pristane or PBS, and the mice were treated with Metformin, INK128 or Rapamycin after 5 months pristane injection. After 2 months treatment, the proportions of G-MDSCs were analyzed by flow cytometry. **(Q)**The percentage of G-MDSCs in Lung, bone marrow, PECs and Spleen. The statistical graphs are shown. Data represent the mean ± SEM. **P*≤0.05, ** *P*≤0.01, *** *P* ≤0.001.

**Figure S2．The mRNA expression among SLE pathway was markedly different in MDSCs in Pristane-induced lupus mice.**

After the Pristane-induced lupus mouse model was established, G-MDSCs and M-MDSCs were purified from Spleen-derived MDSCs mice using Myeloid-Derived Suppressor Cell Isolation Kit. Then, the whole-genome transcriptome profiling were performed by RNA sequencing. **(A)** The mRNA expression changes of M-MDSCs between control and lupus mice was shown in the volcano plot. **(B)** The pathway enrichment analysis of differentially expressed mRNAs in M-MDSCs is shown. The figure was generated using the R language tool. **(C)** The differential expression of a set of mRNAs in SLE pathway in M-MDSCs from control and lupus mice is shown in the heat map. **(D)** The mRNA expression changes of G-MDSCs between control and lupus mice was shown in the volcano plot. **(E, F)** The pathway enrichment analysis of differentially expressed mRNAs in G-MDSCs is shown. The figure was generated using the R language tool. **(G)** The differential expression of a set of mRNAs in SLE pathway in M-MDSCs from control and lupus mice is shown in the heat map.

**Figure S3．TLR7/IFN-αsignal pathway is an important in the differential mRNA in both M-MDSCs and G-MDSCs between lupus mice and control mice were clustered in the PPI network by the string database.** After the Pristane-induced lupus mouse model was established, G-MDSCs were purified from Spleen-derived MDSCs mice using Myeloid-Derived Suppressor Cell Isolation Kit. Then, the whole-genome transcriptome profiling were performed by RNA sequencing. **(A)**The differential mRNA in M-MDSCs between lupus mice and control mice were clustered in the PPI network by the string database. **(B)** The differential mRNA in G-MDSCs between lupus mice and control mice were clustered in the PPI network by the string database.

**Figure S4. The percentage of M-MDSCs and G-MDSCs in tissues.** BALB/c mice (10 weeks, n=6-8/group) were given a single intraperitoneal injection of 0.5 ml pristane or PBS and monitored for 7 months. Metformin treated lupus mice for 2 months after 5 months pristane injection. The percentage of M-MDSCs and G-MDSCs in bone marrow (BM) (A), Spleen (B), Kidney (C). (I)The statistical graphs are shown. Data represent the mean ± SEM. *P≤0.05, ** P≤0.01, *** P ≤0.001.

**Figure S5. mTOR inhibitor INK128 can reverse the abnormal expression of surface molecules and SLE related molecules on G-MDSCs and M-MDSCs.**

BALB/c mice were given a single intraperitoneal injection of 0.5 ml pristane or PBS and the mice treated with INK128 at 5th month and monitored at 7th month. M-MDSCs and G-MDSCs are isolated from spleens of each group. MDSCs (G-MDSCs or M-MDSCs) from three mice in the same group were mixed. Next, differential gene expressions in each group (control group, lupus groups, INK128-treated group) are analyzed by RNA sequencing. **(A)** INK128 could reverse the abnormal mRNA expression (208 decreased mRNAs and 648 increased mRNAs; *P*<0.05) of M-MDSCs in lupus mice. **(B)** INK128 could reverse the abnormal mRNA expression (370 decreased mRNAs and 213 increased mRNAs; *P*<0.05) of G-MDSCs in lupus mice. **(C)** By GO analysis, we analyzed the differences in surface molecular expression of G-MDSCs among the groups. INK128 could reverse the abnormal surface molecules (GO:001620, *P*<0.05) mRNA expression in G-MDSCs in lupus mice. The data is shown in the heat map. **(D)** The SLE signal–related molecules (PATH ID:05322) identified by pathway analysis on G-MDSCs and G-MDSCs in mice with pristane-induced lupus. INK128 could reverse the abnormal SLE related mRNA expression in G-MDSCs in lupus mice. The data is shown in the heat map. **(E)** By GO analysis, we analyzed the differences in surface molecular expression of G-MDSCs among the groups. INK128 could reverse the abnormal surface molecules (GO:001620, *P*<0.05) mRNA expression in M-MDSCs in lupus mice. The data is shown in the heat map. **(F)** The SLE signal–related molecules (PATH ID:05322, *P*<0.05) identified by pathway analysis on M-MDSCs and G-MDSCs in mice with pristane-induced lupus. INK128 could reverse the abnormal SLE related mRNA expression in M-MDSCs in lupus mice. The data is shown in the heat map.

**Figure S6. AMPK/mTOR signal pathway regulates G-MDSCs differentiation *in vitro*. (A)** Mouse BM cells were induced to MDSCs by adding GM-CSF (40ng/ml) and IL-6 (40ng/ml) and were treated with various concentrations of INK128 (0-100nM). Percentage of G-MDSCs were determined by flow cytometry. **(B)** Mouse BM cells were treated with 50nM INK128 with different days and were induced to MDSCs. The proportions of G-MDSCs were analyzed by flow cytometry. **(C)** Mouse BM cells were induced to MDSCs by adding GM-CSF (40ng/ml) and IL-6 (40ng/ml) and were treated with various concentrations of Metformin (0-5mM). Percentage of G-MDSCs were determined by flow cytometry. **(D)** Mouse BM cells were treated with 2mM INK128 with different days and were induced to MDSCs. The proportions of G-MDSCs were analyzed by flow cytometry. **(E)** Mouse BM cells were cultured for 4 days in GM-CSF and IL-6 with or without Metformin INK128, R848, IFN-α. Percentages of M-MDSCs were detected. Results were expressed as mean±SD of three independent experiments. **P*≤0.05, ** *P*≤0.01, *** *P*≤0.001.

**Figure S7.** **The transcription factor IRF-8 is crucial for G-MDSCs differentiation.** After the Pristane-induced lupus mouse model was established, G-MDSCs were purified from Spleen-derived MDSCs mice using Myeloid-Derived Suppressor Cell Isolation Kit. Then, the whole-genome transcriptome profiling were performed by RNA sequencing. **(A)** The differential mRNA expression of hematopoietic cell lineage pathway in G-MDSCs between lupus mice and control mice are shown. **(B)** The relationship of IRF-8 with the differential mRNA expression in G-MDSCs are shown in Co-expression network relation map. **(C)** Mouse BM cells were cultured for 4 days in GM-CSF and IL-6 with or without Metformin, R848 and IFN-α. The proportions of G-MDSCs were analyzed by flow cytometry.

**Figure S8.** **The expression levels of p-AMPK, p-mTOR and IRF-8 in kidney in each group.** BALB/c mice (10 weeks, n=6-8/group) were given a single intraperitoneal injection of 0.5 ml pristane or PBS and monitored for 7 months. Metformin or INK128 treated lupus mice for 2 months after 5 months pristane injection. Immunohistochemistry assay was applied to detect the expression levels of p-AMPK**(A)**, p-mTOR**(B)** and IRF-8 **(C)**in kidney in each group. Images were obtained on a microscope of Olympus BX43. **(D)** Integrated optical density (IOD) and the area of target distribution were measured by Image-Pro Plus 6.0 software. We calculated the mean density of each field and took the average of the mean density of five fields as the mean density of each case.

**Figure S9. The differential expression of microRNAs in G-MDSCs from spleens of lupus mice and control mice is shown in the heat map.** Relative quantitation of *miR-143-3p, miR-451a, miR-199a-3p, miR-199a-5p, miR-144-3p, miR-143-5p, miR-547-3p,* and *miR-199b-5p* expressions by RT-PCR in G-MDSCs. Results were expressed as mean±SD of three independent experiments. **P*≤0.05, ** *P*≤0.01, *** *P*≤0.001.
