# Supplementary material for: IRF-8/miR-451a regulates M-MDSC differentiation via the AMPK/mTOR signal pathway during lupus development
Source: Cell Death Discov. 2021 Jul 16;7:179. doi: 10.1038/s41420-021-00568-z (PMC8289825; doi:10.1038/s41420-021-00568-z)
Supplement: Supplementary file 3 — Supplementary material 2 [file 41420_2021_568_MOESM3_ESM.docx]

| **Gene** | **F/R** | **Sequence** |
| --- | --- | --- |
| ***IRF-8*** | F | 5^,^ TGGAAGCATCCACCTCCTGATTGT 3^,^ |
|  | R | 5^,^ TGATCGAACAGATCG ACAGCAGCA 3^,^ |
| ***CEBP-β*** | F | 5^,^ AAGCTGAGCGACGAGTACAAGA 3^,^ |
|  | R | 5^,^ GTCAGCTCCAGCACCTTGTG 3^,^ |
| ***Rb-1*** | F | 5^,^ CAGGGCTGTGTTGACATCGGAGTA 3^,^ |
|  | R | 5^,^ CCACGGGAAGGACAAATCTGTTC 3^,^ |
| ***CEBP-α*** | F | 5^,^ GCGGGAACGCAACAACATC 3^,^ |
|  | R | 5^,^ GTCACTGGTCAACTCCAGCAC 3^,^ |
| ***PU-1*** | F | 5^,^ CCT CCA TCG GAT GAC TTG GT 3^,^ |
|  | R | 5^,^ ATG GTG TGC GGA GAA ATC CC 3^,^ |
| ***GAPDH*** | F | 5^,^ GGTGAAGGTCGGTGTGAACG 3^,^ |
|  | R | 5^,^ CTCGCTCCTGGAAGATGGTG 3^,^ |
| ***miR-143-3p*** | F | 5^,^ GACCGCTGAGATGAAGCACTGT 3^,^ |
|  | R | 5^,^ GTGCAGGGTCCGAGGTATTC 3^,^ |
| ***U6*** | F | 5^,^ GCTTCGGCAGCACATATACT 3^,^ |
|  | R | 5^,^ GTGCAGGGTCCGAGGTATTC 3^,^ |
| ***miR-451a*** | F | 5^,^ ACACTCCAGCTGGGAAACCGTTACCATTACT 3^,^ |
|  | R | 5^,^ CTGGTGTCGTGGAGTCGGCAA 3^,^ |
| ***miR-199a-3p*** | F | 5^,^ ACACTCCAGCTGGGACAGTAGTCTGCACAT 3^,^ |
|  | R | 5^,^ TGGTGTCGTGGAGTCG 3^,^ |
| ***miR-199a-5p*** | F | 5^,^ GTG CTC ACC CAG TGT TCA GAC 3^,^ |
|  | R | 5^,^ TAT GGT TGT TCT GCT CTC TGT CTC 3^,^ |
| ***miR-144-3p*** | F | 5^,^ CCCTACAGTATAGATGATG 3^,^ |
|  | R | 5^,^ TGCAGGGTCCGAGGT 3^,^ |
| ***miR-143-5p*** |  | 5^,^ GGGGTGAGATGAAGCACTG 3^,^ |
|  |  | 5^,^ CAGTGCGTGTCGTGGAGT 3^,^ |
| ***miR-199b-5p*** |  | 5^,^ GCCCGCCCAGTGTTT AGACTAT 3^,^ |
|  |  | 5^,^ GTGCAGGGTCCGAGGT 3^,^ |
