# Supplementary figures and images for: IRF-8/miR-451a regulates M-MDSC differentiation via the AMPK/mTOR signal pathway during lupus development
Source: Cell Death Discov. 2021 Jul 16;7:179. doi: 10.1038/s41420-021-00568-z (PMC8289825; doi:10.1038/s41420-021-00568-z)

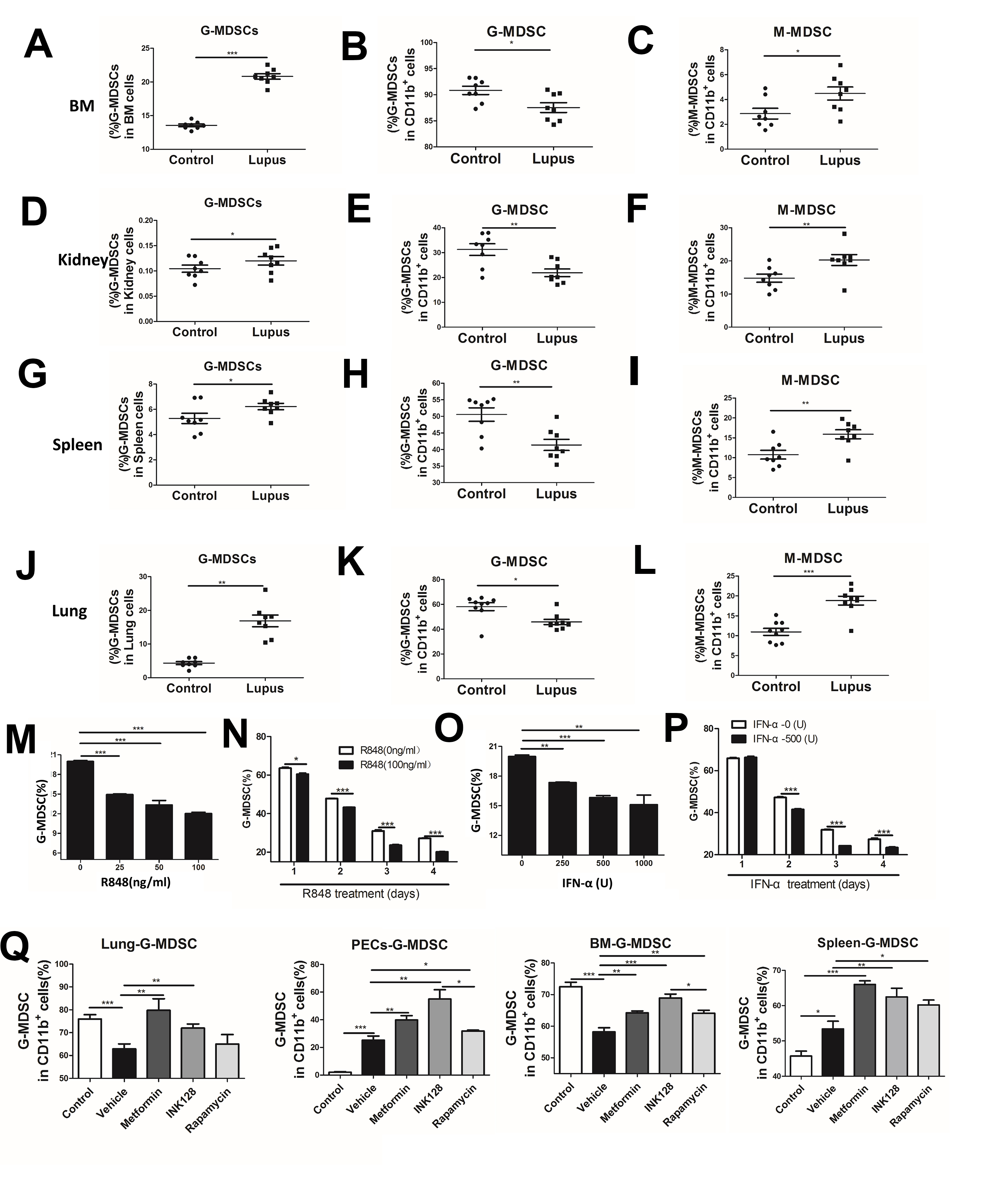

Supplement: Supplementary file 4 — FigureS1 [file 41420_2021_568_MOESM4_ESM.png]

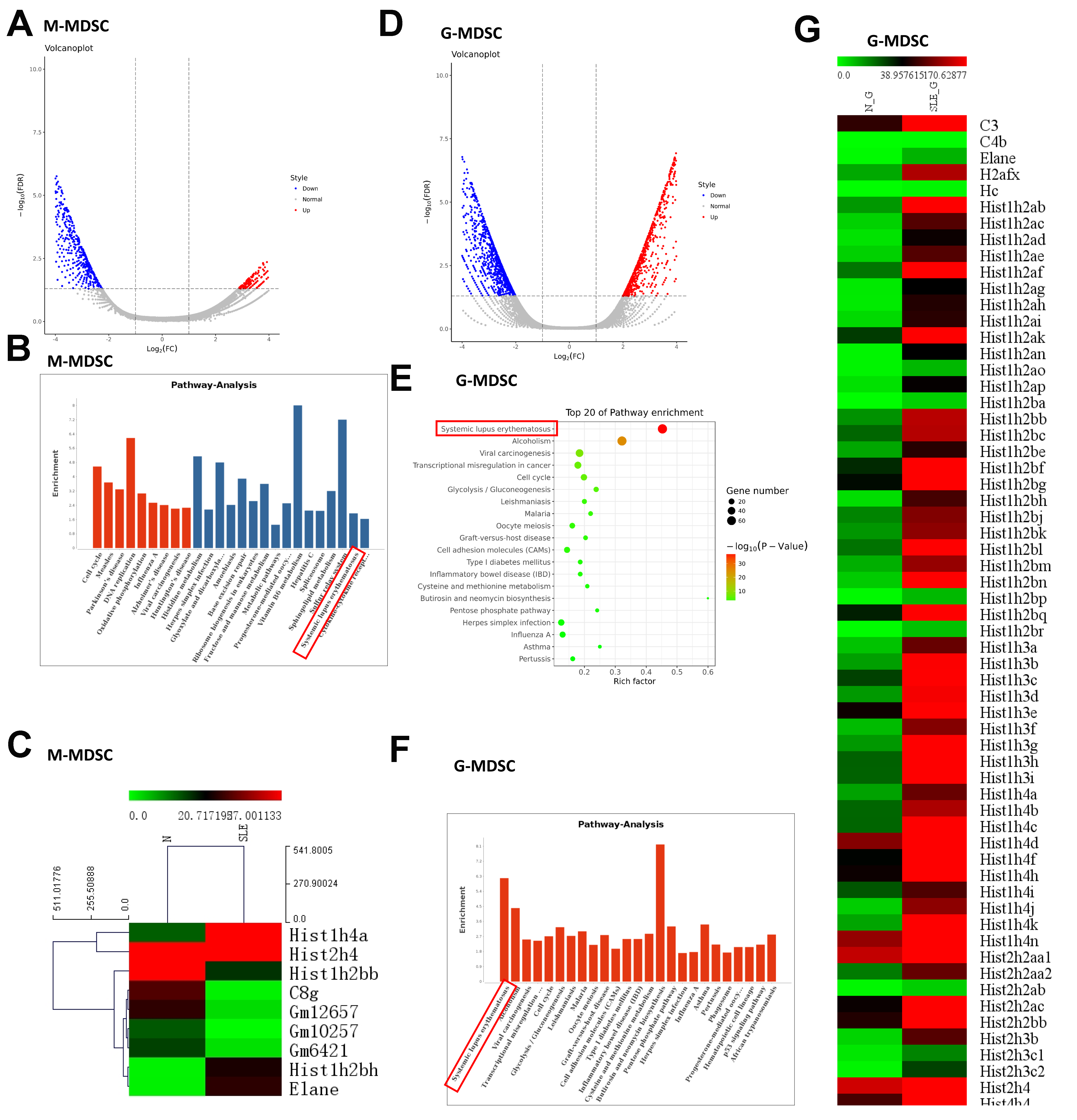

Supplement: Supplementary file 5 — FigureS2 [file 41420_2021_568_MOESM5_ESM.png]

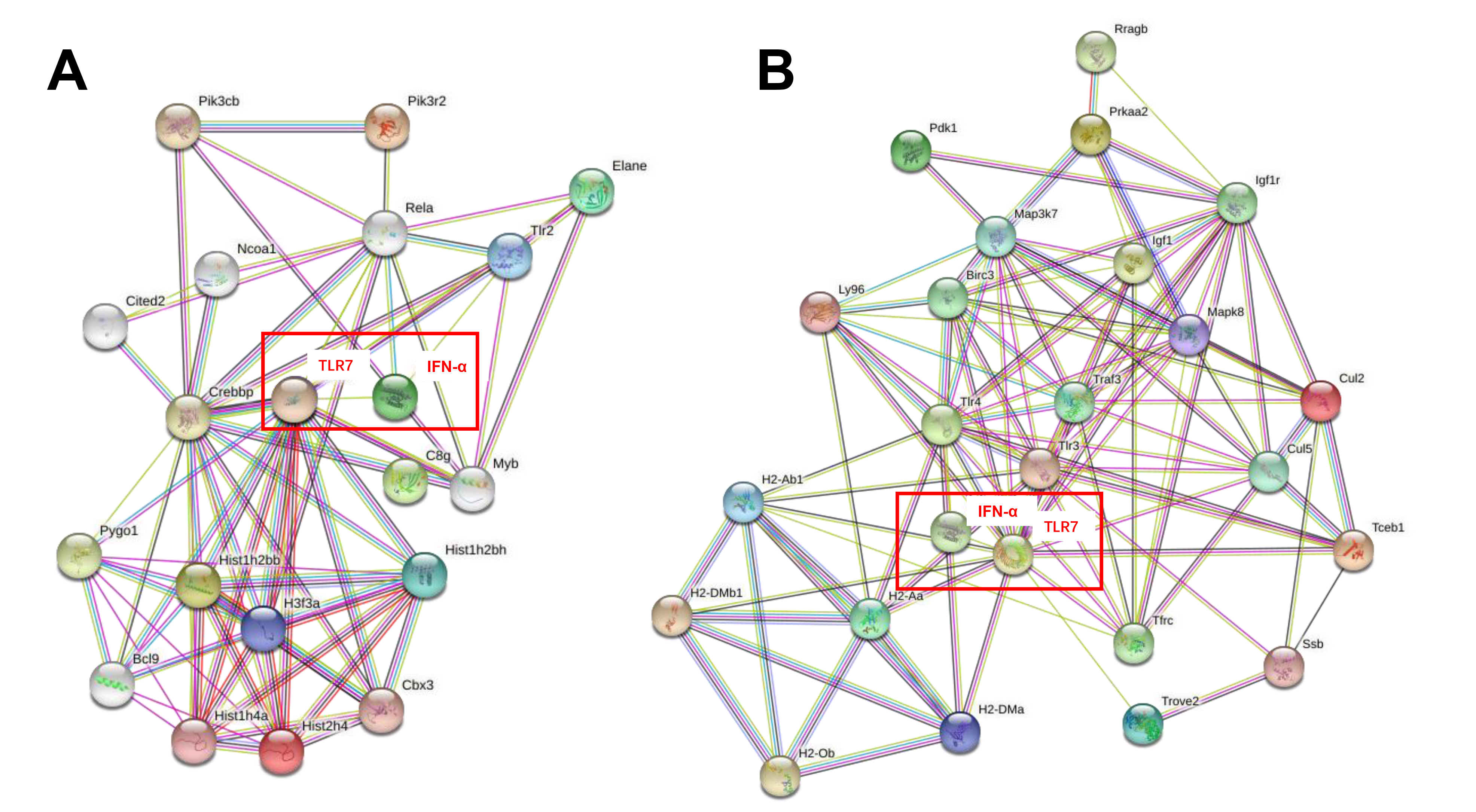

Supplement: Supplementary file 6 — FigureS3 [file 41420_2021_568_MOESM6_ESM.png]

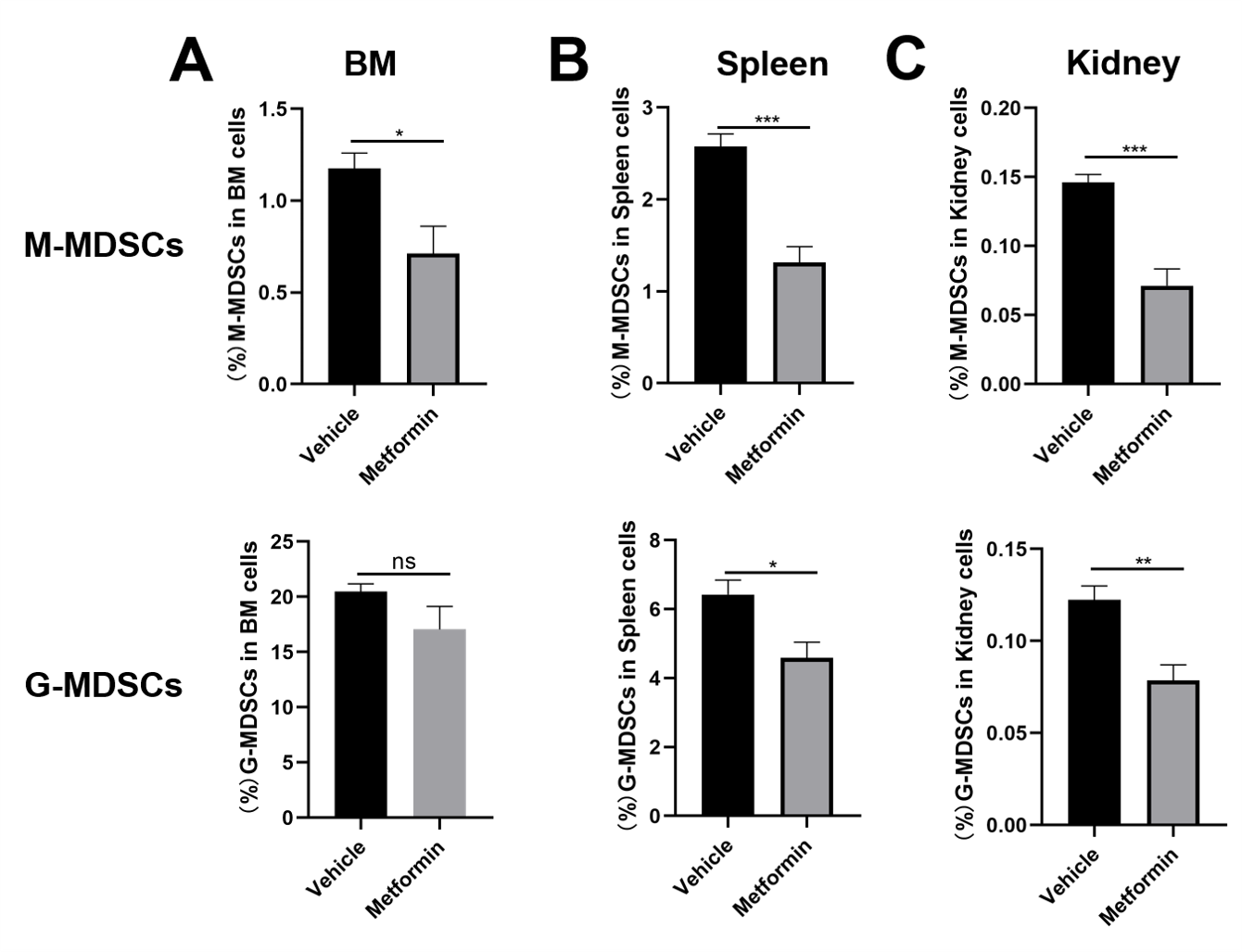

Supplement: Supplementary file 7 — FigureS4 [file 41420_2021_568_MOESM7_ESM.png]

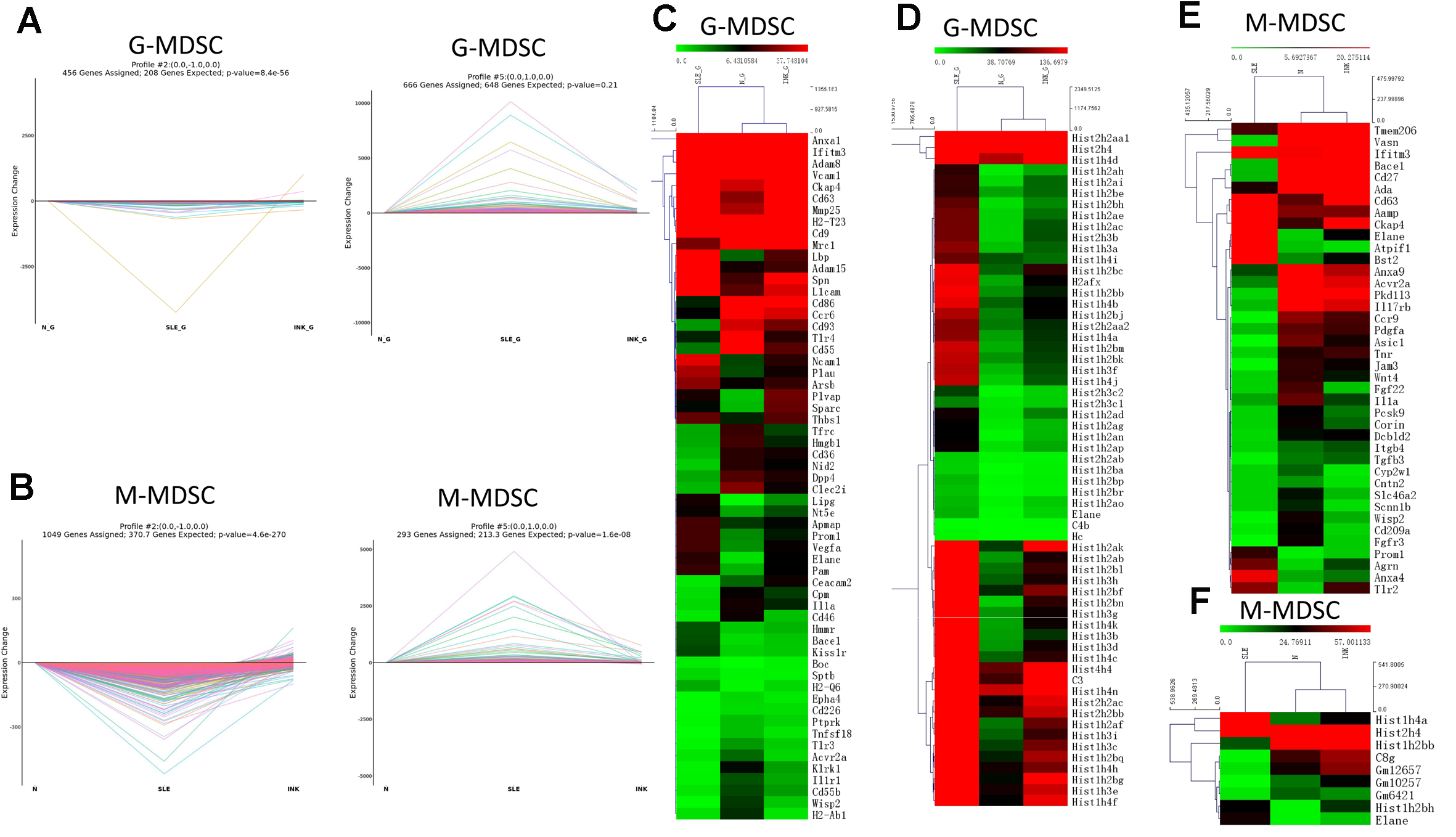

Supplement: Supplementary file 8 — FigureS5 [file 41420_2021_568_MOESM8_ESM.png]

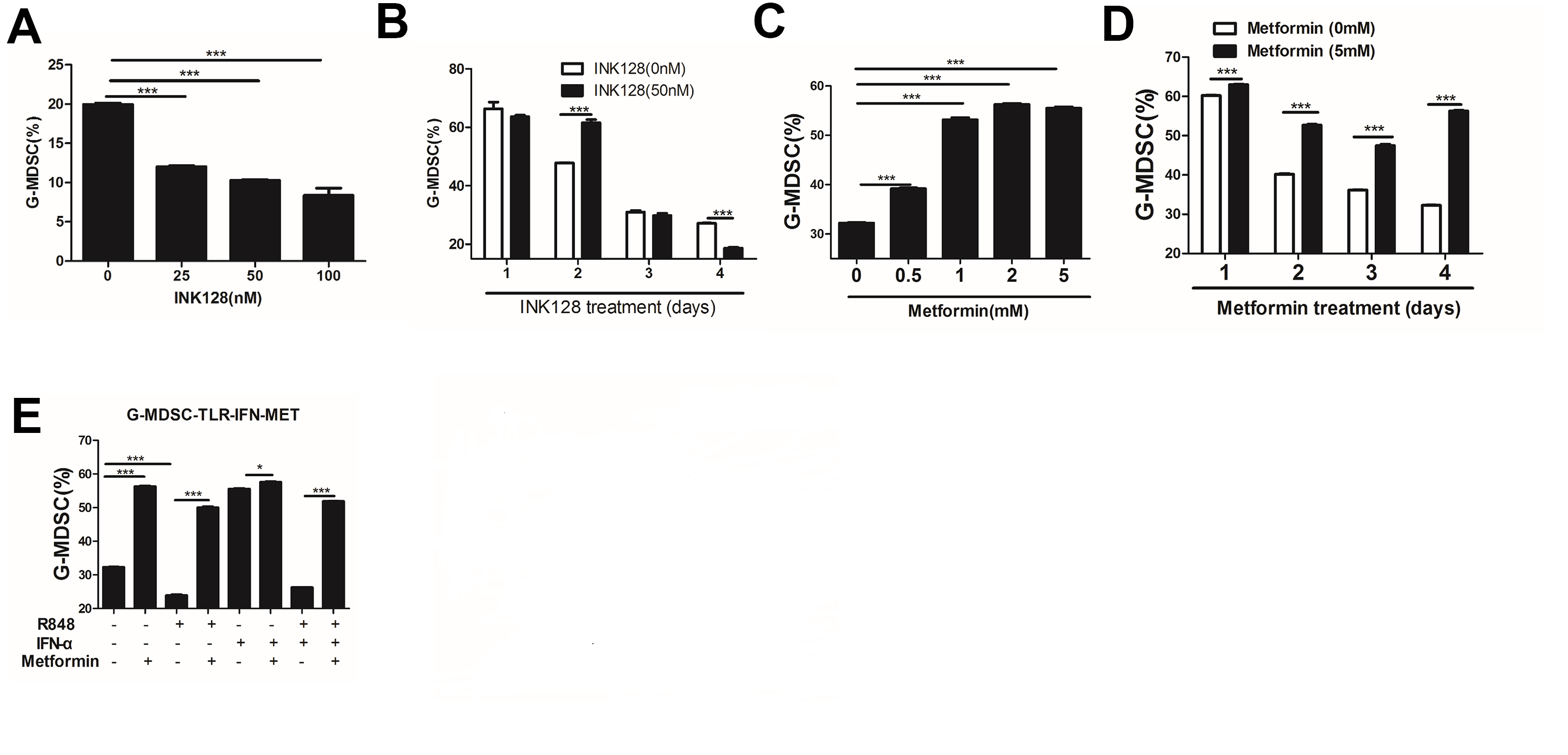

Supplement: Supplementary file 9 — FigureS6 [file 41420_2021_568_MOESM9_ESM.png]

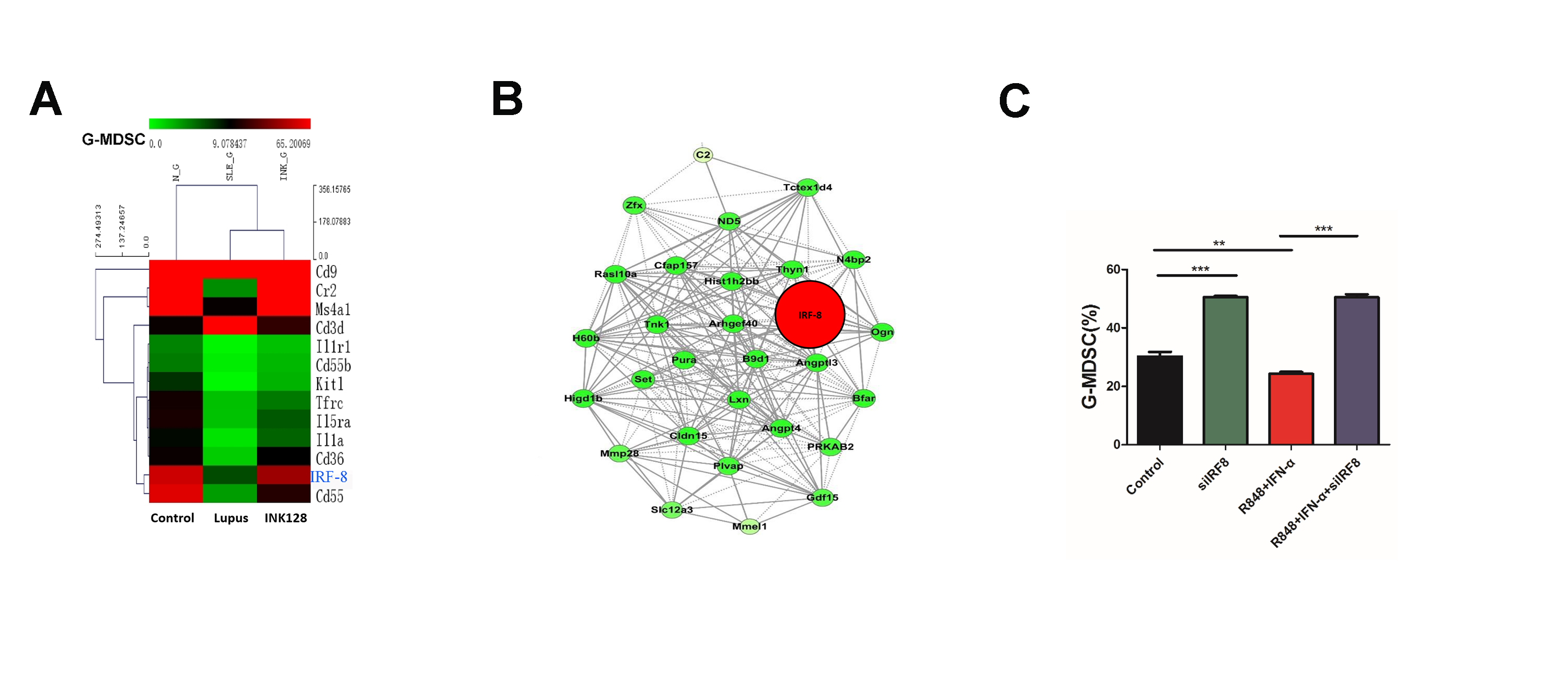

Supplement: Supplementary file 10 — FigureS7 [file 41420_2021_568_MOESM10_ESM.png]

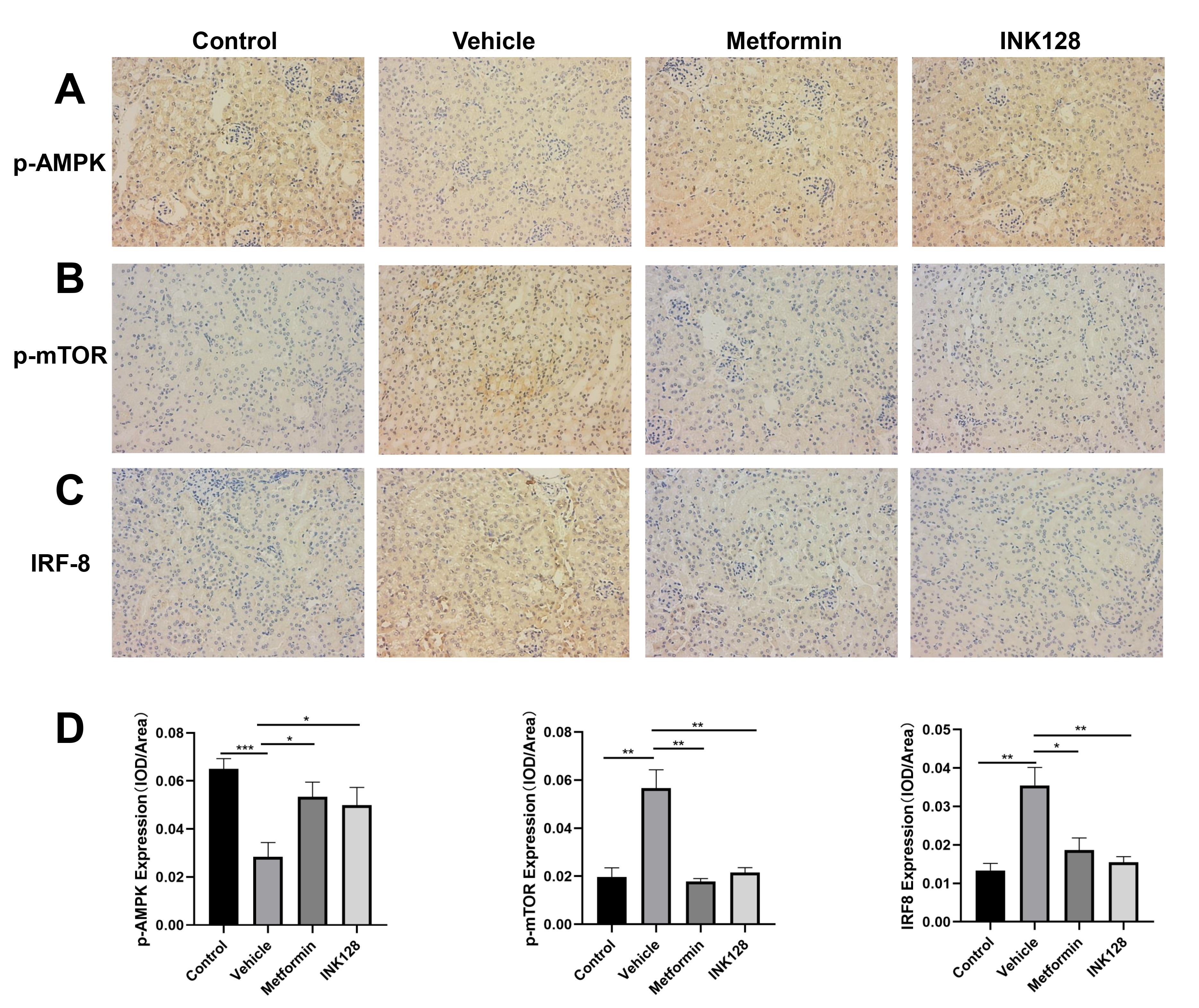

Supplement: Supplementary file 11 — FigureS8 [file 41420_2021_568_MOESM11_ESM.png]

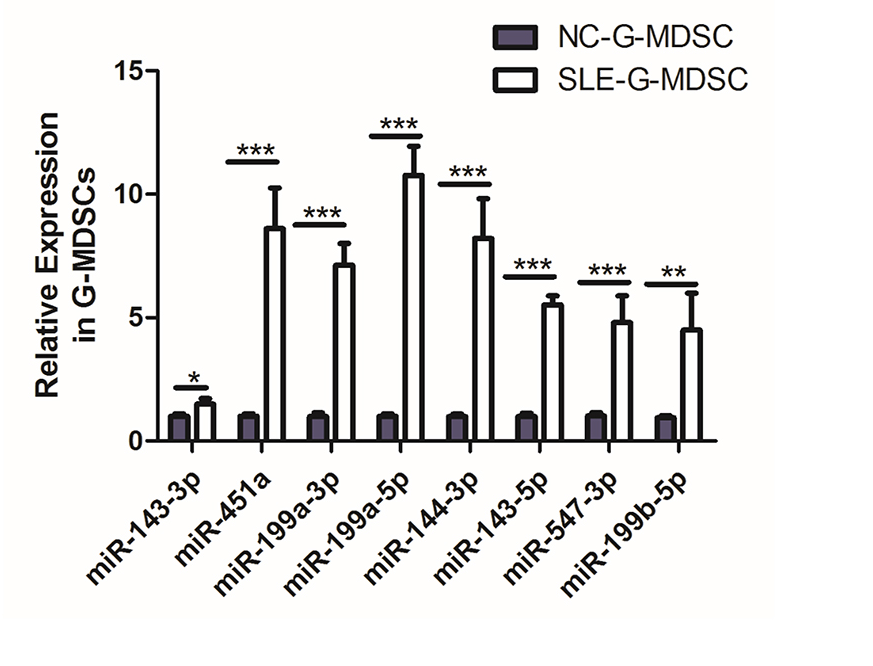

Supplement: Supplementary file 12 — FigureS9 [file 41420_2021_568_MOESM12_ESM.png]
